# Integrated PacBio SMRT and Illumina sequencing uncovers transcriptional and physiological responses to drought stress in whole-plant *Nitraria tangutorum*

**DOI:** 10.3389/fgene.2024.1474259

**Published:** 2024-10-01

**Authors:** Meiying Wei, Bo Wang, Chaoqun Li, Xiaolan Li, Cai He, Yi Li

**Affiliations:** College of Forestry, Gansu Agricultural University, Lanzhou, China

**Keywords:** *Nitraria tangutorum* Bobr., drought stress, transcriptome analysis, plant hormone signal transduction, whole plant

## Abstract

**Introduction:**

*Nitraria tangutorum* Bobr., a prominent xerophytic shrub, exhibits remarkable adaptability to harsh environment and plays a significant part in preventing desertification in northwest China owing to its exceptional drought and salinity tolerance.

**Methods:**

To investigate the drought-resistant mechanism underlying *N. tangutorum*, we treated 8-week-old seedlings with polyethylene glycol (PEG)-6000 (20%, m/m) to induce drought stress. 27 samples from different tissues (leaves, roots and stems) of *N. tangutorum* at 0, 6 and 24 h after drought stress treatment were sequenced using PacBio single-molecule real-time (SMRT) sequencing and Illumina RNA sequencing to obtain a comprehensive transcriptome.

**Results:**

The PacBio SMRT sequencing generated 44,829 non-redundant transcripts and provided valuable reference gene information. In leaves, roots and stems, we identified 1162, 2024 and 232 differentially expressed genes (DEGs), respectively. The Kyoto Encyclopedia of Genes and Genomes (KEGG) analysis revealed that plant hormone signaling and mitogen-activated protein kinase (MAPK) cascade played a pivotal role in transmitting stress signals throughout the whole *N. tangutorum* plant following drought stress. The interconversion of starch and sucrose, as well as the biosynthesis of amino acid and lignin, may represent adaptive strategies employed by *N. tangutorum* to effectively cope with drought. Transcription factor analysis showed that *AP2/ERF-ERF, WRKY, bHLH, NAC* and *MYB* families were mainly involved in the regulation of drought response genes. Furthermore, eight physiological indexes, including content of proline, hydrogen peroxide (H2O2), malondialdehyde (MDA), total amino acid and soluble sugar, and activities of three antioxidant enzymes were all investigate after PEG treatment, elucidating the drought tolerance mechanism from physiological perspective. The weighted gene co-expression network analysis (WGCNA) identified several hub genes serve as key regulator in response to drought through hormone participation, ROS cleavage, glycolysis, TF regulation in *N. tangutorum*.

**Discussion:**

These findings enlarge genomic resources and facilitate research in the discovery of novel genes research in *N. tangutorum*, thereby establishing a foundation for investigating the drought resistance mechanism of xerophyte.

## 1 Introduction

Drought is one of the most severe abiotic stresses worldwide that poses a significant threat to the survival and development of plants (Osmolovskaya et al., 2018). Due to the sessile nature, plants are subjected to endure drought stress and have evolved a sophisticated array of response mechanisms through long-term evolution. Once upon drought stress, plants perceive signals by various signaling transduction (Hirayama and Shinozaki, 2010; Takahashi et al., 2018). Plant hormones, including abscisic acid (ABA), auxin (IAA), cytokinin (CTK), gibberellin (GA), ethylene (ET), brassinosteroid (BR), jasmonic acid (JA) and salicylic acid (SA), have been identified for their involvement in the signaling networks as signal molecules (Lamaoui et al., 2018; Waadt, 2020). The mitogen-activated protein kinase (MAPK) cascade is also a stress-responsive signal transduction pathway that becomes activated in plants upon perceiving drought. MAPK cascade is involved in signal transduction between cells as well as within and outside the cell nucleus (Chen et al., 2023). Besides, due to the role as a second messenger in transducing extracellular stimuli into the intracellular environment, Ca^2+^ signaling also plays a pivotal role as a crucial signaling pathway in the drought response (Bartels and Sunkar, 2005). Along with signal transduction processes, different transcription factors (TF) like *AP2/ERF*, *WRKY*, *NAC*, *bHLH,* and *MYB*, are involved in the plant’s response to drought, and play essential roles through regulating the expression of downstream genes by binding with the *cis*-elements (Joshi et al., 2016). A series of drought-responsive genes will be activated, leading to the reprogramming the metabolism and growth of plant, resulting in various physiological and metabolic changes, such as the accumulation of reactive oxygen species (ROS) and activation of antioxidant enzymes (Li M. et al., 2022), dynamic interconversion between starch and sugars (Dong and Beckles, 2019), metabolism of amino acid and phenylpropanoid (Batista-Silva et al., 2019; Diniz et al., 2020; Dong and Lin, 2021). Ultimately, plants respond to drought at the whole-plant level through gene regulation and physiological adaptability. In conclusion, multiple signaling transduction pathways, TFs regulation and metabolic adjustments synergistically respond to drought stress in plants (Yang F. and Lv G., 2022).

Understanding the mechanisms of plant drought resistance is crucial for investigating strategies to mitigate the adverse effects of drought on plants and for cultivating genetically modified crops with enhanced drought tolerance through genetic technology (Ullah et al., 2017; Singh et al., 2015). The transcriptomic analysis has been extensively employed for elucidating the underlying mechanisms of plant drought resistance. The Illumina RNA-seq technique enables accurate quantification of genes alteration among tissues based on temporal and spatial expression profiling in many plants (Chen et al., 2021a). However, the effectiveness of reconstructing and annotating is hindered by the constraint imposed by short read. The SMRT sequencing provides the advantage of generating long and full-length transcripts, but it may be prone to inaccuracies (Li W. L. et al., 2021; Zhao et al., 2018). Therefore, the integration of Illumina RNA-seq with SMRT-seq can synergistically result in comprehensive transcriptional information for various plants species. The approach has been practiced in many studies, such as *Haloxylon* (Yang F. and Lv G. H., 2022), *Populus ussuriensis* (Li W. et al., 2021), *Pinus massoniana* L. (Mei et al., 2020) and *Actinidia valvata* (Li Z. et al., 2022).

*Nitraria tangutorum* Bobr. is a super-xerophytic shrub that falls under the Zygophyllaceae family, primarily found in the northwestern region of China (Zhu et al., 2021). As a desert plant, *N. tangutorum* possess easily produced branches, small leaves and extensive root system, showing strong adaptability to abiotic challenges like drought, high salinity, alkali condition, and sand burial in harsh environments. It is capable of thriving in arid and semiarid desert environments, where the average annual precipitation ranges from 140.9 to 302.2 mm (Zheng et al., 2014). These characteristics makes it an outstanding candidate species for investigating drought-stress tolerance mechanism. However, the research on *N. tangutorum* has mainly focused on its physiological responses to various stimuli (Gao et al., 2022; Didi et al., 2022; Yang et al., 2010a; Yang et al., 2010b), the identification of bioactive compounds in its fruit, leaves and seeds (Chen et al., 2021b; Hu et al., 2014; Jiang et al., 2021; Zhou et al., 2019), and a few transcriptome sequencing studies with the absence of whole-genome data (Liu C. et al., 2022; Liu et al., 2023; Wang et al., 2015; Wang et al., 2022; Zhu et al., 2021). Liu et al. have identified the genes and metabolic pathways in response to drought stress through RNA-sequencing by taking leaf tissues (Liu C. et al., 2022). But the drought response mechanism of each tissue in *N. tangutorum* with the prolongation of drought stress lacks comprehensive and systematic transcriptome data.

In light of the deficiencies in current research on drought resistance mechanisms of *N. tangutorum*, our objective is to address three key issues: How do the tissues of *N. tangutorum* respond to drought stress? What is the molecular mechanism underlying its drought resistance? Which key genes are involved in *N. tangutorum*’s response to drought? To find the answers, we conducted physiological analyses and transcriptome comparison in three tissues of *N. tangutorum* under drought stress stimulated by polyethylene glycol (PEG) 6,000 at 0 h, 6 h, and 24 h, using an integration of full-length and Illumina RNA sequencing data. The dynamics gene expression in each tissue, proposed potential molecular mechanisms and key genes underlying drought tolerance of *N. tangutorum* were investigated. The findings will enrich genetic resources underlying research on drought-tolerance and facilitate exploration of the novel genes for breeding plants with enhanced drought resistance.

## 2 Materials and methods

### 2.1 Plant materials and drought treatment

The *N. tangutorum* seeds were acquired from Nuomuhong farm, Haixi autonomous Prefecture, Qinghai Province in the northwest region of China (96°11′E, 36°33′N). They underwent a 24-h soaking period in sterile water, followed by surface sterilization with ethanol solution (75%, v/v) for 1 min and sterile water for five times. The clean seeds were sown in plastic containers (10 cm × 10 cm × 10 cm) filled with heat-sterilized river sand, containing 12 seeds per container. These containers were placed inside the greenhouse trays at Gansu Agricultural University (103°70′E, 36°09′N) with the temperature of 28°C ± 2°C/26°C ± 2°C (day/night), the photoperiod lasted for 16 h while darkness prevailed for 8 h, the relative humidity was maintained 60% ± 10%. After germination, the seedlings received weekly irrigation using half-strength Murashige and Skoog (MS) medium (Hope, Qingdao, China). The nutrient solution was prepared by dissolving 39.45 g of medium in 1 L of water and carefully pouring into the tray, ensuring complete saturation of the sand in the container. Poor seedlings were removed after 4 weeks of germination.

At the age of 8 weeks, the underperforming seedlings were selectively removed again to ensure the preservation of robust and uniformly sized seedlings. PEG 6,000 solution (20%, m/m) was applied to induce drought stress, in the same ways as irrigating with half-strength MS solution. Different tissues from *N. tangutorum* (roots, stems and leaves) were harvested at 0, 6, and 24 h after treatments (named as R0h, R6h, R24h; S0h, S6h, S24h; L0h, L6h, and L24h) (Supplementary Figure S1–S3). For each time point, three biological replicates were performed for each of the three tissues (3 tissues × 3 time points × 3 biological replications = 27 samples), with each replicate consisting of 12 individual seedlings. Root samples were collected from all seedlings’ root tissues, stem samples were collected from the middle section of the seedlings, approximately 3 cm in length, and leaf samples were taken from the leaves attached to stem samples (Supplementary Figure S4). All the samples immediately stored in liquid nitrogen for 4 h after collection, followed by freezing at −80°C in a refrigerator to facilitate RNA extraction and relevant experiment.

### 2.2 Preparation cDNA library of Illumina RNA-seq, PacBio SMRT-seq and sequencing

The EASYspin Plant microRNA Kit RN40 (Aidlab, Beijing, China) was utilized to extract total RNA from 27 samples. The quality of RNA samples was assessed by a NanoDrop 2000 instrument (Thermo Fisher Scientific, Waltham, MA, United States), while their integrity was evaluated by Agient 2,100 and LabChip GX instruments (PerkinElmer, Waltham, MA, United States). High-quality RNA samples were utilized for cDNA library construction employing the VAHTS Universal V6 RNA-seq Library Prep Kit for Illumina. Subsequently, sequencing of the libraries was performed on Illumina NovaSeq 6,000 platform (Illumina, San Diego, CA, United States) in 150-bp paired-end mode at Biomarker Technologies Corporation (Beijing, China).

To prepare the full-length cDNA library for PacBio SMRT-seq, a pooled sample comprising an equivalent quantity of total RNA from 27 samples was prepared. The SMRTbell library was performed utilizing the SMRTbell Template Prep Kit (Pacific Biosciences, Menlo Park, CA, United States), and the sequencing was established on Sequel II system (Pacific Biosciences, Menlo Park, CA, United States) at Biomarker Technologies Corporation (Beijing, China).

### 2.3 Sequences analysis

The RNA-seq raw data was filtered by fastp (v0.23.2) (Chen et al., 2018) to remove the adapter sequences, reads with over 10% N content, and low-quality reads, the resulting clean data was assembled into unigenes using trinity (v2.5.1) software (Grabherr et al., 2011).

The Iso-Seq3 pipeline (https://github.com/PacificBiosciences/IsoSeq) was utilized to analyze the PacBio raw subreads. Circular consensus (CCS) subreads were obtained from the subreads bam files using CCS (v6.2.0) with a minimum quality threshold set at 0.9 and full passes ≥3 for a zero-mode waveguide (ZMW). By examining the presence of the correct 5′primer, 3′primer, and polyA tail within the CCS sequence, we divided CCSs into full-length non-chimeric reads (FLNC) and non-full-length non-chimeric (nFL) reads. And then Lima (v2.1.0) and isoseq3 (v3.4.0) refine were employed to eliminate poly(A) tails and cDNA primers. High-quality consensus sequences for FLNC transcripts were derived using the ICE clustering algorithm. Classification based on post-correction accuracy exceeding 99% was performed for these high-quality FL consensus sequences. Cd-hit (identity >0.99) was used for removing redundancy of high-quality FL transcripts sequences, and then non-redundant FLNC transcripts were obtained.

Sequence alignment was performed using STAR (v2.5.0b) to align clean reads of RNA-seq with the non-redundant FLNC transcripts and obtain positional information on the transcript, using the non-redundant FLNC transcripts as a reference (Dobin et al., 2013). Kallisto software (v0.46.1) was utilized to compare the reads of Illumina RNA-seq with full-length transcripts and perform direct counting for transcript expression quantification (Bray et al., 2016). FPKM (Fragments Per Kilobase of transcript per Million mapped reads) was employed as a measure to estimate the level of transcript expression.

### 2.4 Identification of simple sequence repeats (SSR), prediction of lncRNA and open reading frame (ORF)

SSR were identified in transcript sequences exceeding 500 bp using MISA software (v1.0) (http://pgrc.ipk-gatersleben.de/misa/). The screening encompassed mono-, di-, tri-, tetra-, penta-, and hexa-nucleotides, representing six types of SSRs, and the corresponding minimum repeat parameters were 10 for mononucleotide, 6 for dinucleotides, and 5 for the other types.

The identification of potential lncRNA candidates was accomplished by integrating four computational methods, namely Coding-non-Coding Index (CNCI), Coding Potential Calculator (CPC), Coding Potential Assessment Tool (CPAT) and Protein family (Pfam) database. LncRNA candidates were selected from the transcripts that exceeded 200 nt in length and contained more than two exons, which were further refined using CPC (Kong et al., 2007), CNCI (Luo et al., 2014), CPAT (Wang et al., 2013) and Pfam. The finally lncRNA results were obtained by using a Venn diagram to compare the predicted lncRNAs from the four methods.

TransDecoder (v5.0.0) (https://github.com/TransDecoder/TransDecoder/releases) were used for ORF prediction (Haas et al., 2013).

### 2.5 Analysis of differentially expressed genes

The DESeq2 (v1.6.3) tool was utilized for identification the differentially expressed genes (DEGs) among sample groups (Anders and Huber, 2010). The criterion was |log2 (fold change) | >1 and FDR (false discovery rate) < 0.01, based on the FPKM values.

### 2.6 Weighted gene co-expression network analysis (WGCNA)

DEGs with FPKM values less than 1 were filtered out, resulting in 3,165 DEGs used for WGCNA analysis using the WGCNA (v1.47) package in R (Langfelder and Horvath, 2008). The weighted network was constructed with unsigned topological overlap matrix. The values of power, minimum number of genes for modules, and minimum height for merging modules are 5, 30, and 0.3045 respectively. Cytoscape (v3.10.2) was used to visualize the network results.

### 2.7 Quantitative real-time PCR (qRT-PCR) analysis

The TUREscript 1st Stand cDNA Synthesis Kit (Aidlab, Beijing, China) was used for performing reverse transcription. The qRT-PCR was conducted using 2 × SYBR^®^ Green Master Mix (DF Biotech., Chengdu, China) on a qTOWER2.2 Quantitative Real-Time PCR Thermal Cyclers (Analytik Jena, Germany). The *18SrRNA* gene from *N. tangutorum* was employed as a reference for normalizing the data. The relative expression of each transcript was determined using the 2^−ΔΔCT^ method (Rao et al., 2013), and FPKM values were used for comparison. Each sample underwent three biological and three technical replicates. The Beacon designer software (v8.14) was used to design specific primers.

### 2.8 Measurement of physiological indexes

All physiological indexes were assessed utilizing commercially accessible kits from Nanjing Jiancheng Bioengineering Institute (Nanjing, China). The contents of H_2_O_2_, soluble sugar (SS), Malondialdehyde (MDA), proline (Pro) and total amino acid content (TAA) were measure by Hydrogen Peroxide assay kit (A064-1-1) (Zhou et al., 2020), Plant soluble sugar content test kit (A145-1-1) (Wang J. et al., 2021), Malondialdehyde (MDA) assay kit (A003-1-2) (Menhas et al., 2022), proline assay kit (A107-1-1) (Menhas et al., 2022) and Total Amino Acid assay kit (A026-1-1) (Sun et al., 2010), respectively. The activities of CAT, SOD and POD (Menhas et al., 2022) were measured by Catalase (CAT) assay kit (A007-1-1), Total Superoxide Dismutase (SOD) assay kit (A001-1-2) and Peroxidase (POD) assay kit (A084-3-1), respectively. All operational procedures were carried out according to the manufacturer’s instructions. Physiological measurements were obtained by averaging data from three biological samples (*n* = 3).

### 2.9 Data analysis

Excel 2021 was used for data collation and analysis. SPSS (v26.0) software was used to perform one-way variance analysis (ANOVA) on physiological indexes. The Duncan test was employed to analyze the means values of the data for statistical significance (*p*-values <0.05). The Venn diagrams were built using the free online tool (https://bioinfogp.cnb.csic.es/tools/venny/index.html). The heatmaps were constructed using BMKCloud (www.biocloud.net) based on the FPKM values of DEGs.

## 3 Results

### 3.1 Overview of SMRT and RNA-seq sequencing data

A total of 23.92G of SMRT sequencing data were generated. 238,525 CCS reads and 188,559 (79.05%) full length non-chimeric (FLNC) reads were produced. Followed that, a total of 75,987 consensus sequences were generated and 99.99% (75,979) of them being recognized as high-quality sequences. Subsequently eliminating redundant reads led to the acquisition of 44,829 non-redundant FLNC transcripts (Supplementary Figure S5).

The Illumina RNA-seq generated a total of 184.60 Gb clean data (27 samples) with each sample containing more than 6.09 Gb. The Q30 percentages exceeded 93.91% and the GC contents exhibited a range of 44.78%–45.71% (Supplementary Table S1). A total of 47,808 unigenes were collected.

### 3.2 Identification of SSRs, prediction of lncRNA and ORF

**FIGURE 1:**
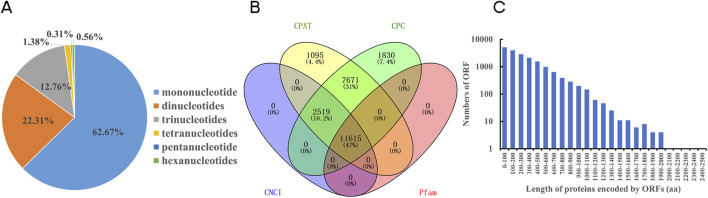
Identification of SSR, prediction of lncRNAs and ORFs. **(A)** Type distribution of SSRs. **(B)** Venn diagram of the predicted lncRNAs. **(C)** The length distribution of protein encoded by ORFs prediction (The vertical axis was scaled logarithmically).

In the study, 21,101 SSRs were identified. Mononucleotides accounts for the largest proportion at 62.67%, followed by dinucleotides (22.31%). Pentanucleotides exhibited the lowest abundance with a ratio of 0.31% (Figure 1A). And totally 11,615 lncRNAs were identified in *N. tangutorum* (Figure 1B). The prediction of ORF and their corresponding amino acid sequences in all non-redundant transcripts sequence was performed. A total of 18,343 complete ORFs were obtained, and the protein encoded by these ORFs had a length distribution ranging from 49aa to 2,427aa (Figure 1C).

### 3.3 Functional annotation of transcripts

**FIGURE 2:**
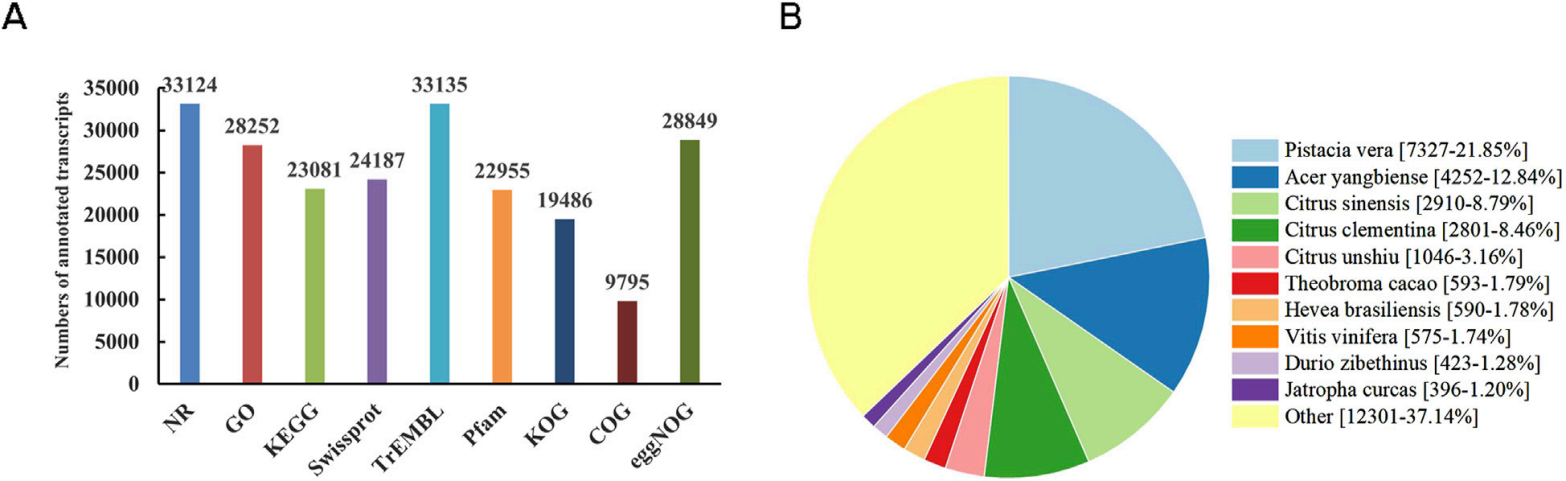
Functional annotation of non-redundant transcripts in *N. tangutorum*. **(A)** Functional annotation of transcripts in nine databases. **(B)** Analysis of the homologous species distribution.

44,829 transcripts were mapped into nine protein and nucleic acid databases by DIAMOND software. Out of the total transcripts, 33,703 (75.2%) obtained annotation information. Among them, 33,124 (98.3%) were annotated in NR (NCBI Non-Redundant protein sequences), 28,252 (83.8%) in GO (Gene Ontology), 23,081 (68.5%) in KEGG (Kyoto Encyclopedia of Genes and Genomes), 24,187 (71.8%) in Swissprot, 33,135 (98.3%) in TrEMBL (Translation of EMBL), 22,955 (68.1%) in Pfam (Protein family), 19,486 (57.8%) in KOG (euKaryotic Orthologous Groups), 9,795 (29.1%) in COG (Clusters of Orthologous Groups), and 28,849 (85.6%) in eggNOG (evolutionary genealogy of genes: Non-supervised Orthologous Groups) (Figure 2A). The identification of homologous species was achieved by aligning transcripts against the NR database. Among them, *Pistacia vera* (7,237, 21.85%) exhibited the highest sequence hits with *N. tangutorum*, followed by *Acer yangbiense* (4,252, 12.84%), *Citrus sinensis* (2,910, 8.79%), *C. clementina* (2,801, 8.46%), *C. unshiu* (1,046, 3.16%), *Theobroma cacao* (593, 1.79%) (Figure 2B).

### 3.4 Identification and KEGG classification of differentially expressed genes (DEGs)

**FIGURE 3:**
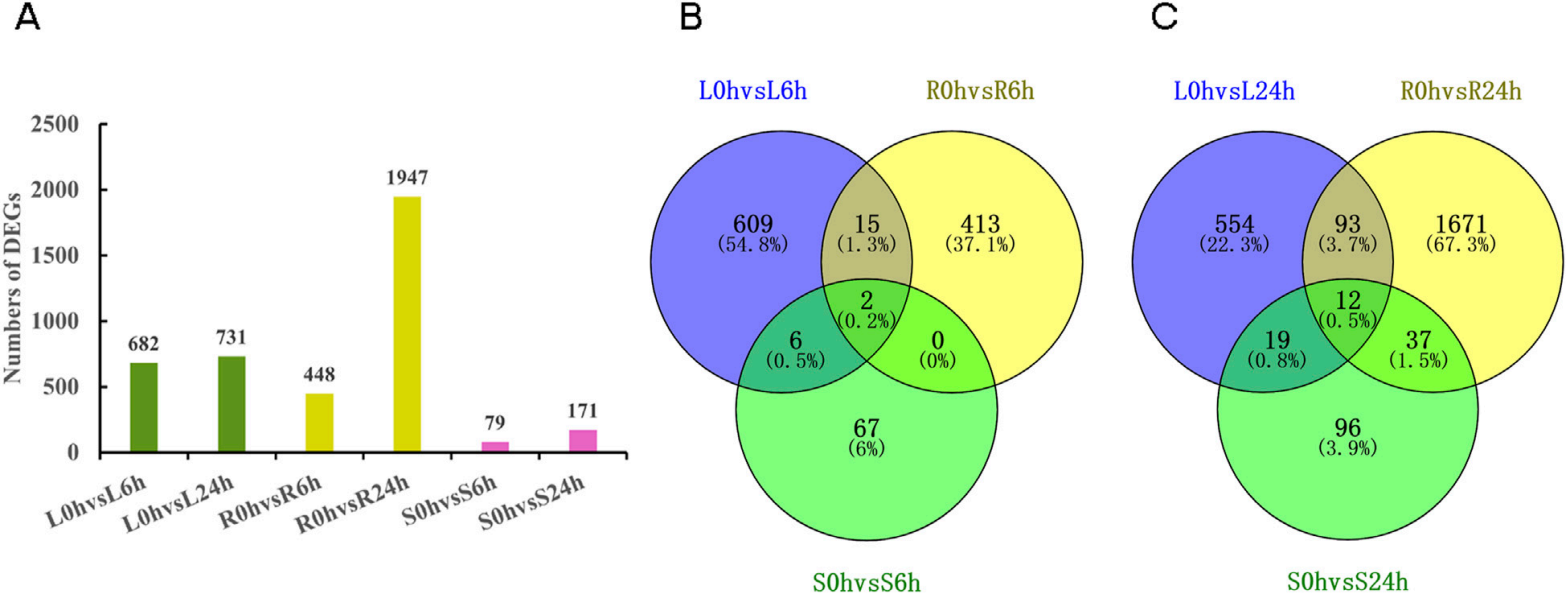
DEGs in *N. tangutorum* under drought stress. **(A)** The number of DEGs in different tissues in 0 h vs. 6 h and 0 h vs. 24 h comparison group (green: DEGs in leaves; yellow: DEGs in roots; fuchsia: DEGs in stems). **(B)** The Venn diagram of annotated DEGs in different tissues in 0 h vs. 6 h comparison groups. **(C)** The Venn diagram of annotated DEGs in different tissues in 0 h vs. 24 h comparison groups.

The comparisons were conducted between the control group at 0 h and different time points of drought stress (6 h, 24 h) in specific tissues. A total of 682 and 731 DEGs were detected in the comparisons between L0h vs. L6h and L0h vs. L24h, 448 and 1,947 DEGs were found in R0h vs. R6h and R0h vs. R24h, 79 and 171 DEGs were identified in S0h vs. S6h and S0h vs. S24h, respectively (Figure 3A). And more than 90% of the DEGs were annotated (Supplementary Table S2). The leaves and roots exhibited the highest number of DEGs at 6 h and 24 h time points in six comparison groups, respectively. The Venn diagram analysis of annotated DEGs showed a limited overlap of DEGs were identified across different tissues following 6 h and 24 h drought treatment (Figures 3B, C). KEGG classification of exclusively expressed DEGs in each tissue at 6 h and 24 h indicated that the predominant clusters were all linked to “metabolic pathways” and “biosynthesis of secondary metabolites” in leaves, roots and stems (Supplementary Figures S6–S11). This implies a highly coordinated response to drought stress at the whole-plant level in *N. tangutorum*.

### 3.5 KEGG analysis of DEGs

**FIGURE 4:**
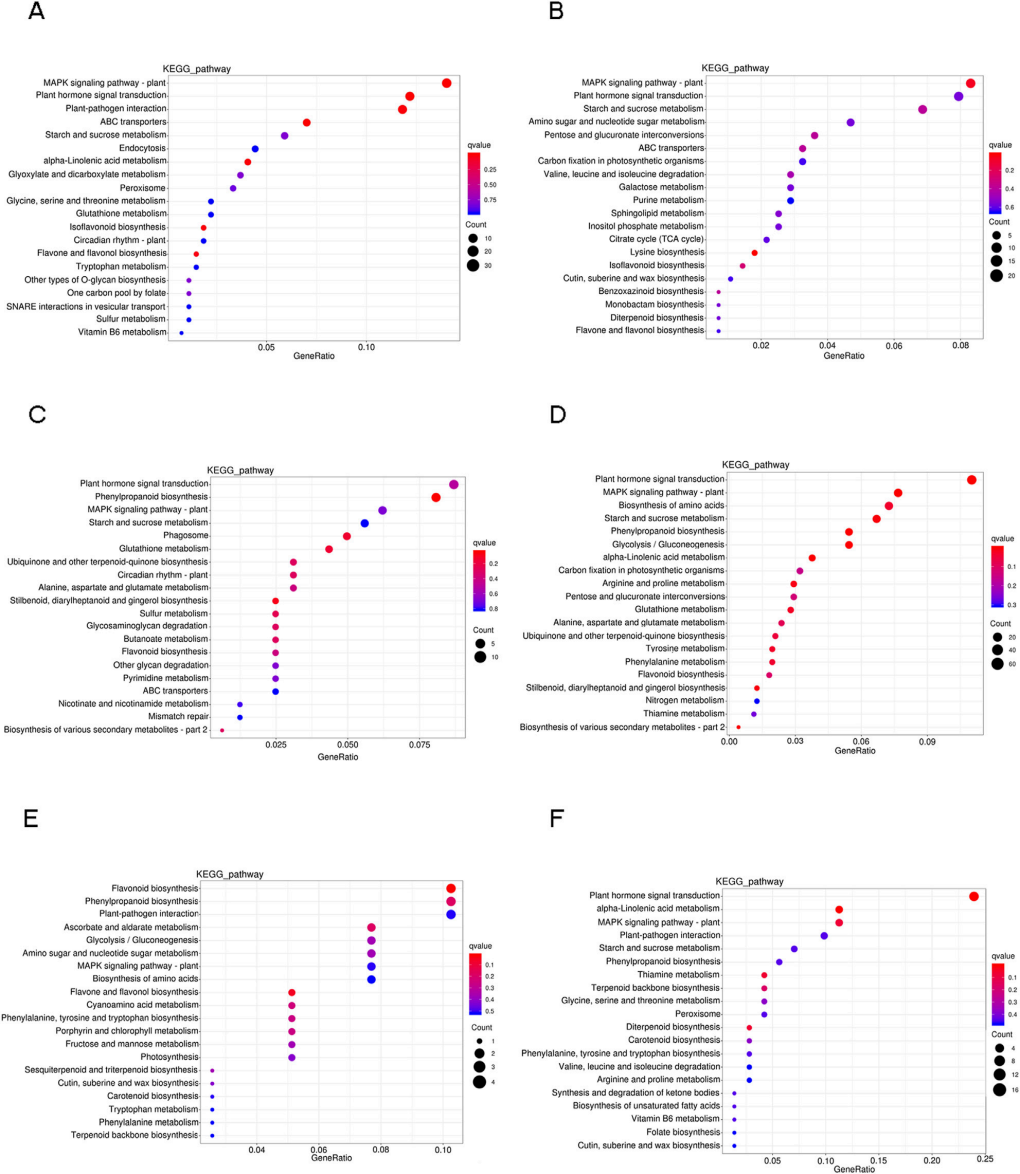
Scatterplot of KEGG pathway enrichment analysis for DEGs in six comparison groups. Only the top 20 pathways were shown. **(A)** L0h vs. L6h. **(B)** L0h vs. L24h. **(C)** R0h vs. R6h. **(D)** R0h vs. R24h. **(E)** S0h vs. S6h. **(F)** S0h vs. S24h.

The DEGs under drought stress were further studied using KEGG pathway enrichment analyses to explore the intricate biological behaviors. A total of 127 pathways exhibited enrichment among the DEGs. The top 20 notable pathways in the leaves, roots and stems were identified. In leaves, the pathways that showed the most significance during the stress process include “MAPK signaling pathway-plant” and “plant hormone signal transduction”. Additionally, at 6 h and 24 h, there is a significant enrichment of the “plant-pathogen interaction” and “starch and sucrose metabolism” pathways respectively (Figures 4A, B). In roots, there was an enrichment of DEGs in pathways related to “plant hormone signal transduction” and “MAPK signaling pathway-plant.” Additionally, “phenylpropanoid biosynthesis,” “starch and sucrose metabolism” and “biosynthesis of amino acids” pathway also showed active representation (Figures 4C, D). In stems, fewer DEGs were found after drought stress. The DEGs were enriched in “flavonoid biosynthesis” in 0 h vs. 6 h group (Figure 4E), while DEGs were dispersedly enriched in “plant hormone signal transduction” and “alpha-linolenic acid metabolism” pathway after 24 h drought stress (Figure 4F).

### 3.6 DEGs associated with plant hormone signal transduction and mitogen-activated protein kinase (MAPK) cascade

**FIGURE 5:**
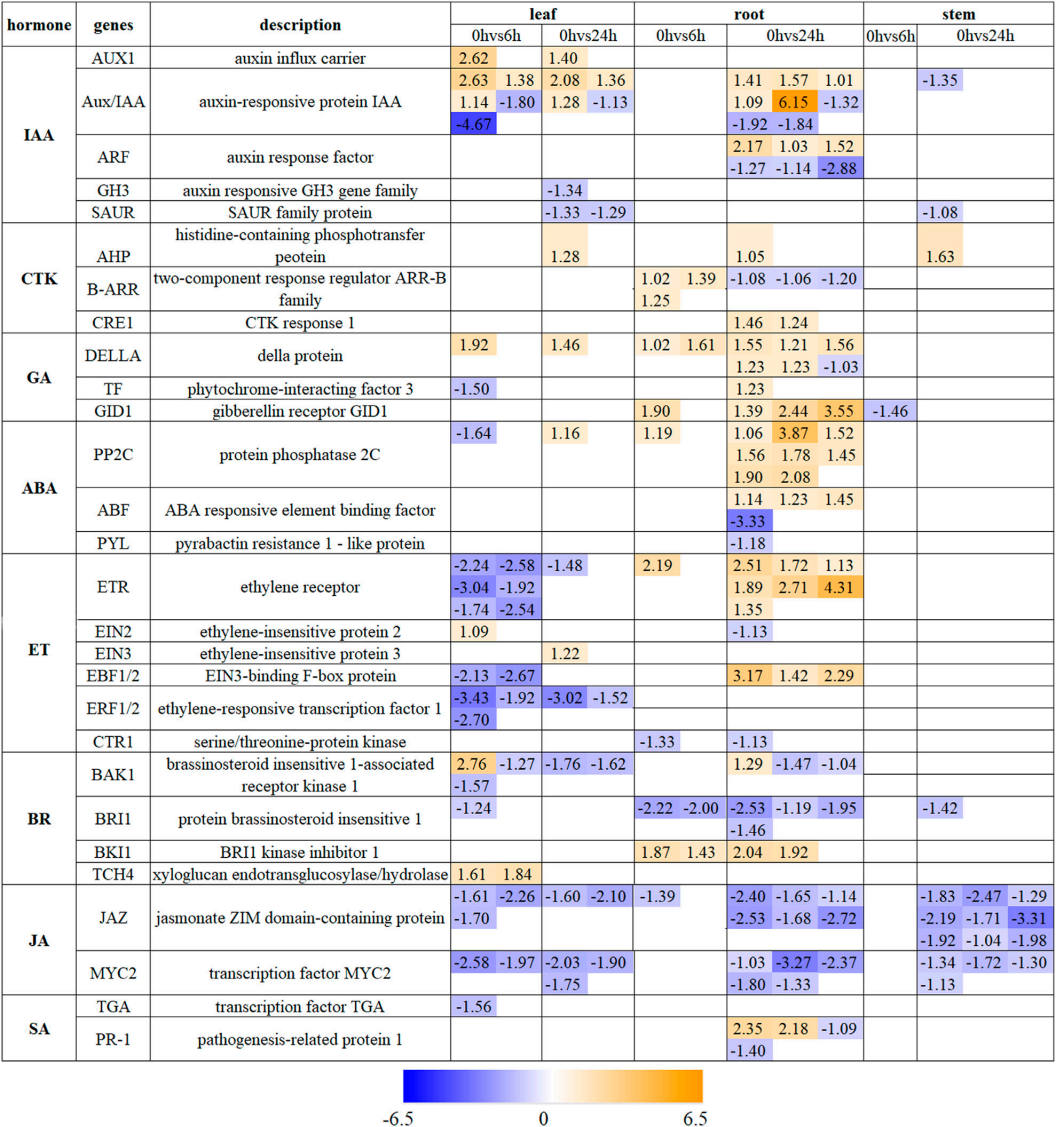
The expression profile of DEGs involved in “Plant hormone signal transduction” in leaves, roots and stems. The number represents the expression changes of DEGs defined as Log_2_FC from upregulation (orange) to downregulation (blue).

The pathway of “plant hormone signal transduction” exhibits a notable enrichment in the whole plant. Hormones that undergo changes in gene expression include IAA, CTK, GA, ABA, ET, BR, JA, and SA (Figure 5). After 6 h drought stress, there were 33 DEGs associated with hormone signaling in leaves, 14 in roots, and 1 in stems. After a period of 24 h under drought conditions, the number changed to 22 DEGs in leaves, 79 in roots and 17 in stems (Figure 5; Supplementary Table S3). All eight hormones mentioned above were related to DEGs found in leaves and roots while only JA was mainly connected with DEGs found in stems.

**FIGURE 6:**
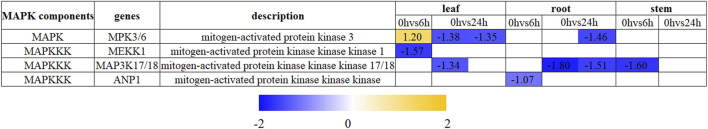
The expression profile of DEGs involved in “mitogen-activated protein kinase (MAPK) cascade” in leaves, roots and stems. The number represents the expression changes of DEGs defined as Log_2_FC from upregulation (orange) to downregulation (blue).

In the present investigation, two components (MAPK and MAPKKK) of the MAPK cascade were found to be DEGs in *N. tangutorum* (Figure 6; Supplementary Table S4). The differential expression of all MAPK genes and the majority of MAPKKK genes in *N. tangutorum* is observed in both leaves and roots, with only one MAPKKK gene showing differential expression in stems.

### 3.7 DEGs associated with the elimination of reactive oxygen species (ROS), starch and sucrose metabolism, phenylpropanoid biosynthesis, amino acid biosynthesis

The current study identified a total of 47 DEGs associated with ROS scavenging. These DEGs included 7 *CAT* genes, 4 *SOD* genes, 17 *POD* genes and 19 glutathione S-transferase (*GST*) genes (Supplementary Table S5). The expression alteration of both *CAT* and *SOD* genes were detected in all three tissues, while *POD* and *GST* genes mainly changed in roots. After 24 h of drought stress, 8 *POD* genes and 14 *GST* genes were upregulated in roots.

**FIGURE 7:**
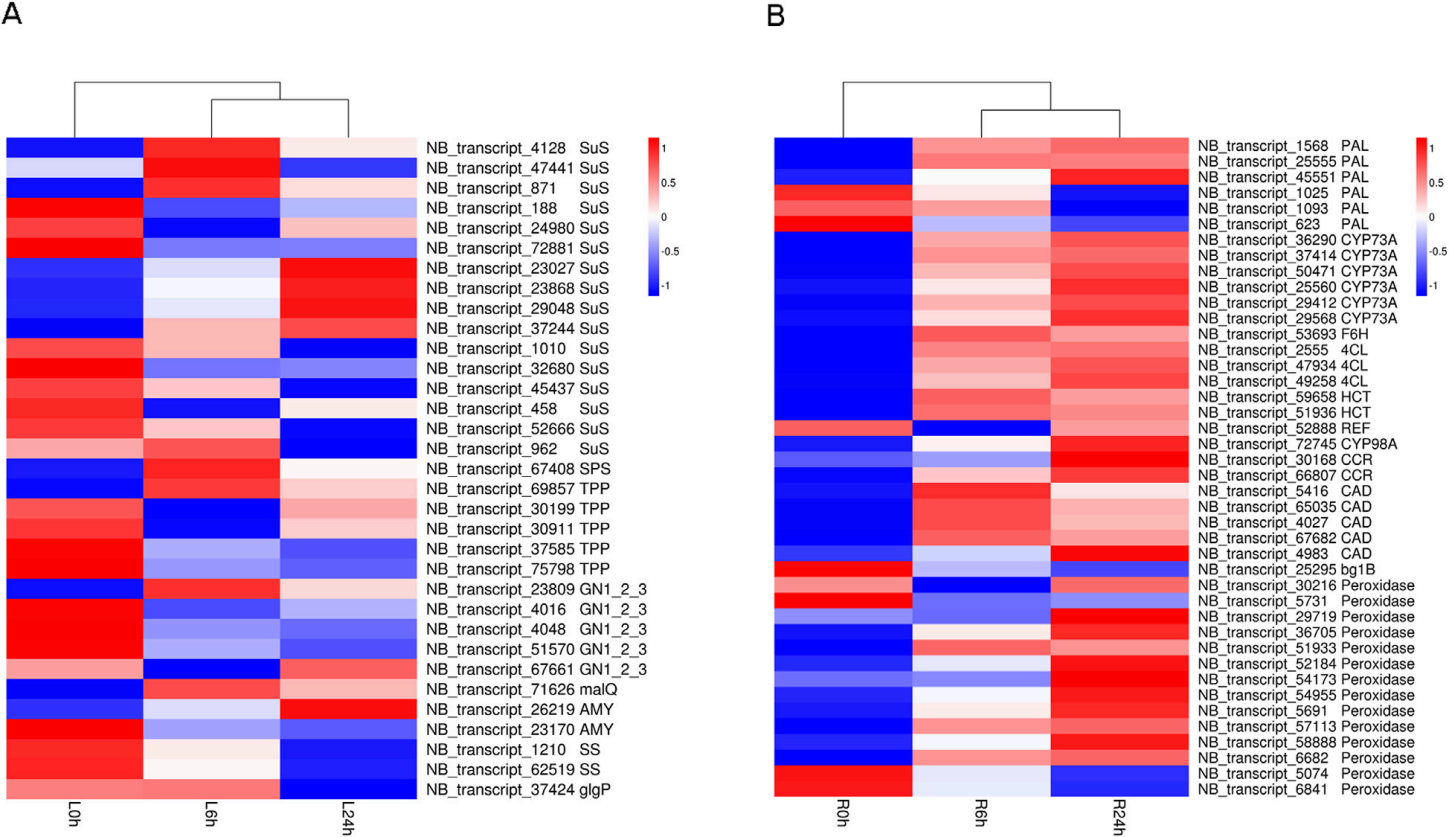
Heatmap of DEGs involved in “starch and sucrose metabolism” pathway and “phenylpropanoid biosynthesis” pathway. **(A)** Heatmap of DEGs in “starch and sucrose metabolism” pathway in leaves. **(B)** Heatmap of DEGs in “phenylpropanoid biosynthesis” pathway in roots.

During the drought stress process at 6 h and 24 h time points, a total of 33 genes were involved in the “starch and sucrose metabolism” pathway in leaves (Figure 7A; Supplementary Table S6). 16 genes encoding sucrose synthase (*SuS*) and 2 genes encoding alpha-amylase (*AMY*) exhibited varying expression patterns. These findings imply the “starch and sucrose metabolism” may play a crucial role in *N. tangutorum*’s ability to cope with drought-induced stress.

The transcriptome analysis revealed that 43 DEGs exhibited enrichment in the “phenylpropanoid biosynthesis” pathway in roots. It was particular noteworthy that the majority of the DEGs were upregulated after being exposed to drought, such as 3 phenylalanine ammonia-lyase (*PAL*) genes, 3 4-coumarate-CoA ligase (*4CL*) genes, 5 cinnamyl-alcohol dehydrogenase (*CAD*) and most of *POD* genes (Figure 7B; Supplementary Table S7). Moreover, these DEGs predominantly reside within the lignin biosynthesis pathway, which constitutes a pivotal branch of the phenylpropanoid biosynthesis (Dong and Lin, 2021).

The analysis revealed that the “biosynthesis of amino acid” pathway in R0h vs. R24h group exhibited enrichment with 52 DEGs. After 24h of drought stress, 44 out of 52 genes were found to be upregulated, including 7 gene encoding delta-1-pyrroline-5-carboxylate synthase (*P5CS*). In the early stage of drought at 6h, only 3 genes showed changes in expression, with one *P5CS* being upregulated (Supplementary Table S8).

### 3.8 Differentially expressed transcription factors (TFs)

**FIGURE 8:**
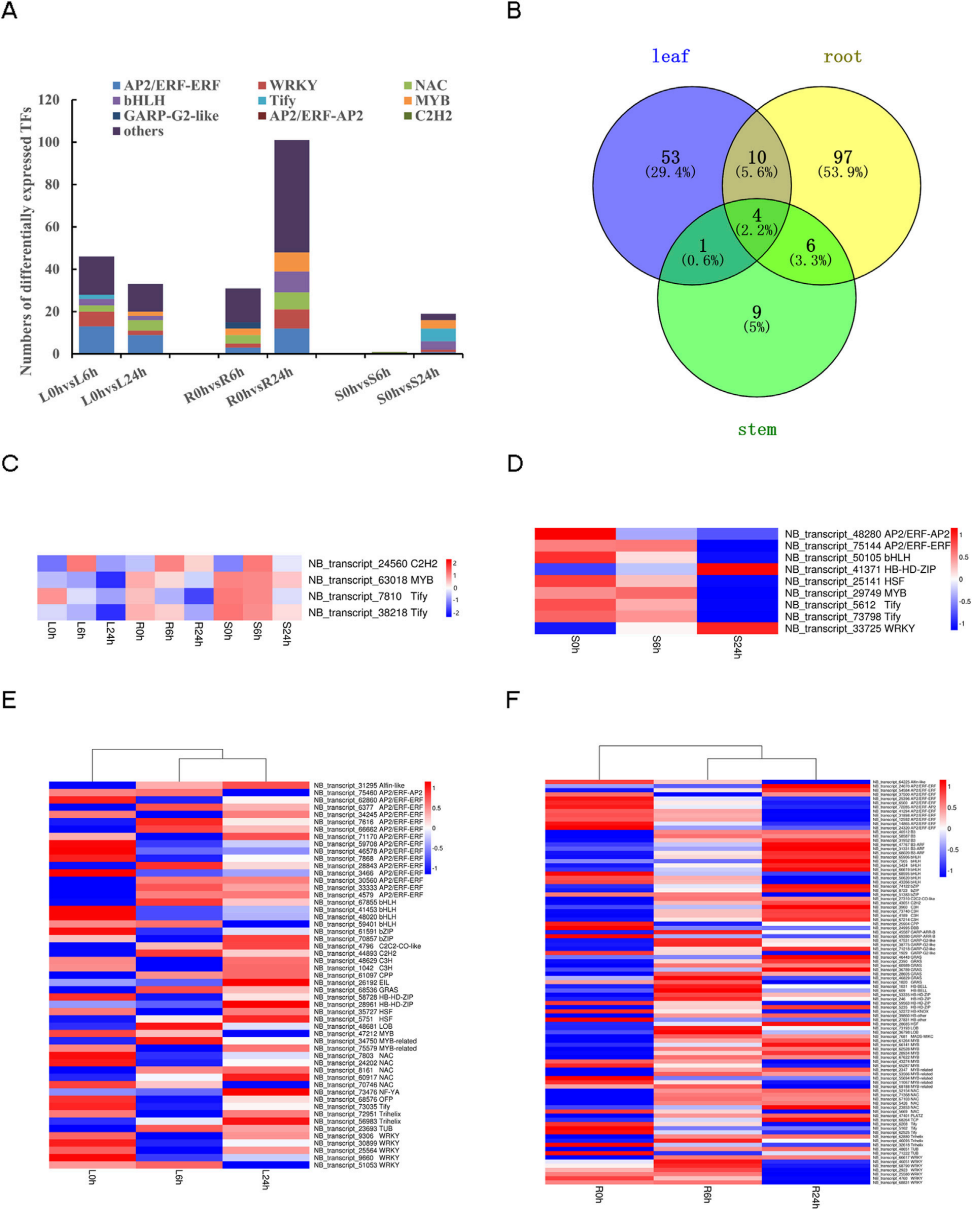
Analysis of differently expressed TFs under drought stress. **(A)** The TFs type composition in all six pairwise comparisons of 0 h, 6 h, 24 h in different tissues. **(B)** The Venn diagram of different expressed TFs in different tissues. **(C)** Heat map of tissue common TFs. **(D)** Heat map of tissue-specific TFs in stems. **(E)** Heat map of tissue-specific TFs in leaves. **(F)** Heat map of tissue-specific TFs in roots.

The present study identified a total of 1,055 transcripts that were predicted to possess TF activity (Supplementary Table S9). The expression of 207 TFs from 35 different families was found to undergo alteration under drought stress conditions (Supplementary Table S10). The 207 TFs mainly enriched in *AP2/ERF-ERF* (32, 15.5%), *WRKY* (21, 10.1%), *bHLH* (16, 7.7%), *NAC* (15, 7.2%) and *MYB* (13, 6.3%) families. The number of differentially expressed TFs identified in pairwise comparisons were L0h vs. L6h (46), L0h vs. L24h (33), R0h vs. R6h (31), R0h vs. R24h (101), S0h vs. S6h (1), S0h vs. S24h (9), respectively (Figure 8A). Additionally, it is noteworthy that leaves, roots, and stems exhibited specific expression of 53, 97, and 9 TFs, respectively (Figure 8B). Most TFs showed differential expression only in one or two tissues. Notably, the expression levels of the 4 specific TFs, *NB_transcript_24560* (*C2H2*), *NB_transcript_63018* (*MYB*), *NB_transcript_7810* (*Tify*), *NB_transcript_38218* (*Tify*), changed across all three tissues (Figure 8C). Heat maps depicting tissue-specific TFs expression pattern revealed differences within or across tissues for TFs belonging to the same gene family (Figures 8D–F).

### 3.9 Physiological indexes of *N. tangutorum* under drought stress

**FIGURE 9:**
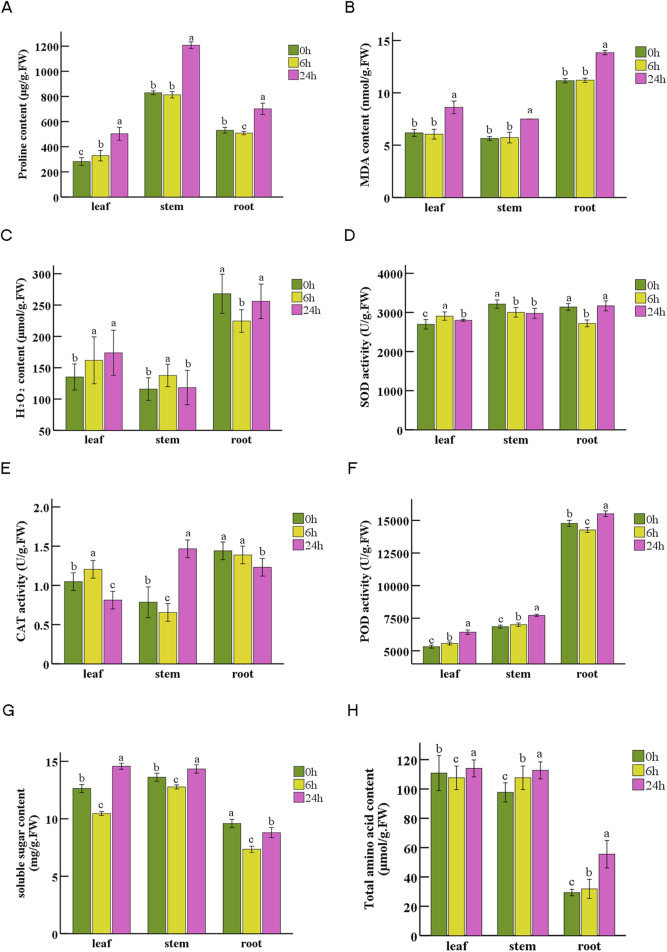
Physiological indexes of *N. tangutorum* in leaves, stems and roots at 0 h, 6 h, 24 h under drought stress. **(A)** Proline content. **(B)** Malondialdehyde (MDA) content. **(C)** H_2_O_2_ content. **(D)** Superoxide dismutase (SOD) activity. **(E)** Catalase (CAT) activity. **(F)** Peroxidase (POD) activity. **(G)** Soluble sugar content. **(H)** Total amino acid content. The data are shown as averages ±SD, n = 3. Different letters indicate the statistically significant differences at *p* < 0.05.

We conducted measurement on eight physiological parameters to examine the impact of drought stress induced by PEG-6000 on *N. tangutorum* at the physiological level (Figure 9). After 6 h of drought stress, a slight increase in leaves proline content was observed, accompanied by minor changes in stems and roots. However, after 24 h drought stress, the proline content exhibited a significant rise of 78.4%, 45.6%, and 32.0% for leaves, stems, and roots respectively compared to the initial measurement at 0 h (Figure 9A). The content of MDA remained relatively stable after 6 h of drought stress, but increased significantly after 24 h. Compared with the content at 0h, it increased by 39.5%, 33.0%, and 24.1% in leaves, stems, and roots respectively (Figure 9B). Regarding ROS-related indexes, the H_2_O_2_ content followed different patterns of change in three tissues: it gradually increased in leaves, increased at 6 h and decreased at 24 h in stems, while roots showed an opposite trend compared to stems (Figure 9C). The activities of SOD, CAT and POD exhibited diverse trends after PEG-stimulated drought stress among tissues at different time point (Figures 9D–F). Additionally, POD activity in roots was 2.56, 2.04 times of that after 6 h and 24 h drought stress in leaves, and 2.41, 2.01 times of that after 6 h and 24 h drought stress in stems (Figure 9F). The soluble sugar (SS)content exhibited a decline at 6 h followed by an upward trend at 24 h after drought stress across all tissues (Figure 9G). The total amino acid (TAA) content remained unchanged during the entire duration of the treatment period for leaves and stems (including 6 h treatment in root), but significantly increased by 89.1% compared to initial level in roots after 24 drought treatment (Figure 9H).

### 3.10 Weighted gene co-expression network analysis (WGCNA)

**FIGURE 10:**
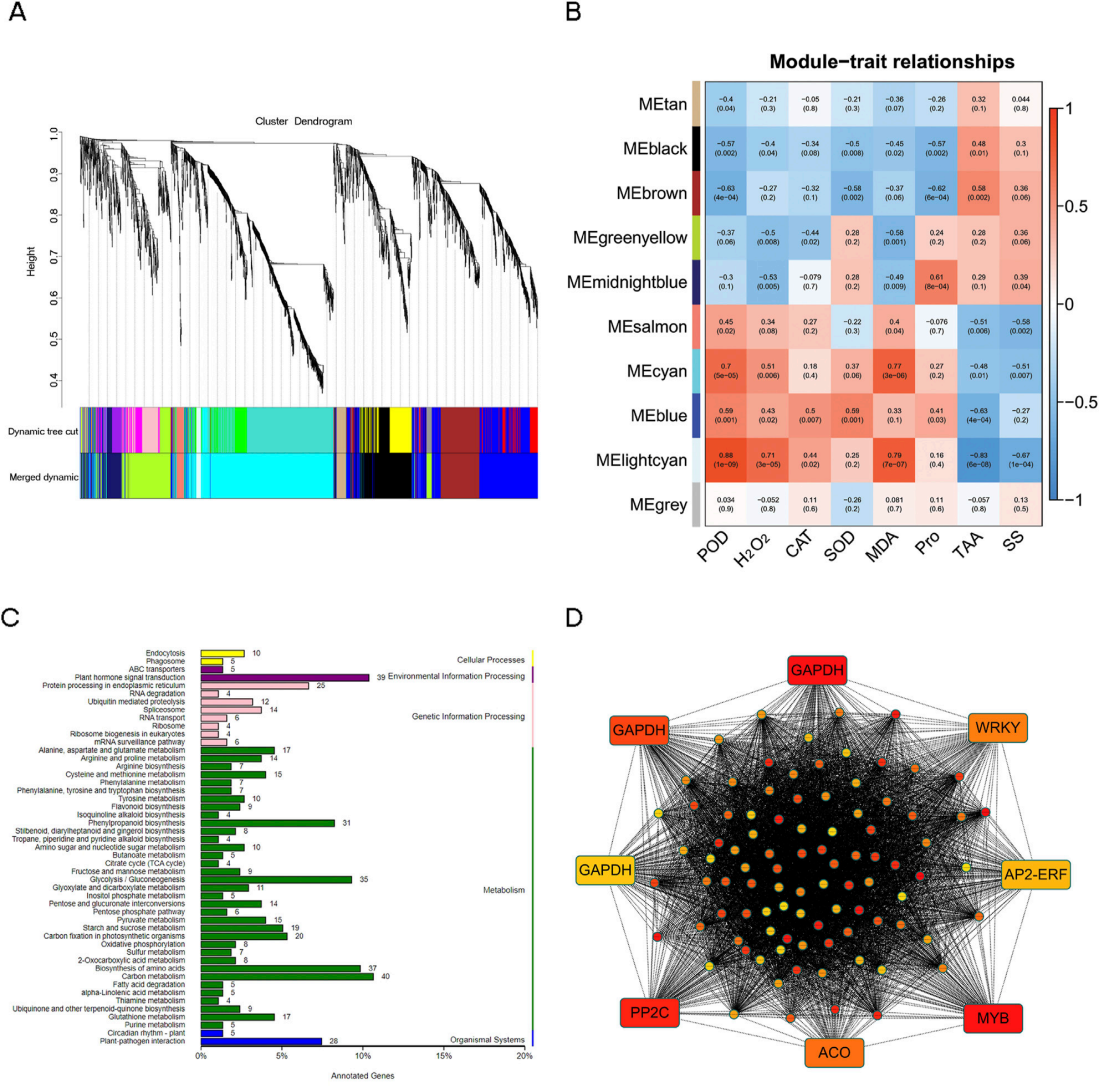
Weighted gene co-expression network analysis. **(A)** Clustering dendrogram of genes with original module (Dynamic Tree Cut) and merged module (Merged Dynamic). **(B)** Module-trait relationships between modules and physiological indexes. The numbers represent the correlation coefficient, and the numbers in parentheses mean *p*-value. **(C)** The KEGG pathway classification of the genes in cyan modules. **(D)** Gene network of the cyan module based on the top 100 hub genes.

WGCNA was conducted to screen the modules and key genes associated with the drought tolerance of *N. tangutorum*. A total of 3,165 DEGs with FPKM>1 were screened for WGCNA analysis. Eight physiological indexes, including POD, H_2_O_2_, CAT, SOD, MDA, Pro, TAA, and SS were assessed as traits to evaluate the relationship among trait and modules (Figure 10A). Among the 10 identified modules, the number of genes ranged from a minimum of 37 (lightcyan) to a maximum of 1,062 (cyan) (Figure 10B; Supplementary Table S11). The cyan module displayed positive correlation with MDA, POD and H_2_O_2_ (correlation coefficients: 0.77, 0.7 and 0.51). The genes in cyan module were primarily enriched in “carbon metabolism,” “plant hormone signal transduction,” “biosynthesis of amino acid” and “glycolysis/gluconeogenesis” (Figure 10C). We selected the top 100 hub genes from the cyan module to construct a gene co-expression network. According to the annotation results of the 100 hub genes, there were 9 of the hub genes remain unannotated in neither of the nine databases in Section 3.3 (Supplementary Table S12), suggesting a substantial scope for further exploration in novel gene research within *N. tangutorum* underlying drought stress. Three genes encoding transcription factors (*MYB*, *WRKY*, *AP2/ERF*), one 1-aminocyclopropane-1-carboxylate oxidase 1 (*ACO*) gene, two glutathione S-transferase (*GST*) genes, three glyceraldehyde-3-phosphate dehydrogenases (*GAPDH*) genes were involved in the hub (Figure 10D).

### 3.11 Quantification and verification of gene expression level

**FIGURE 11:**
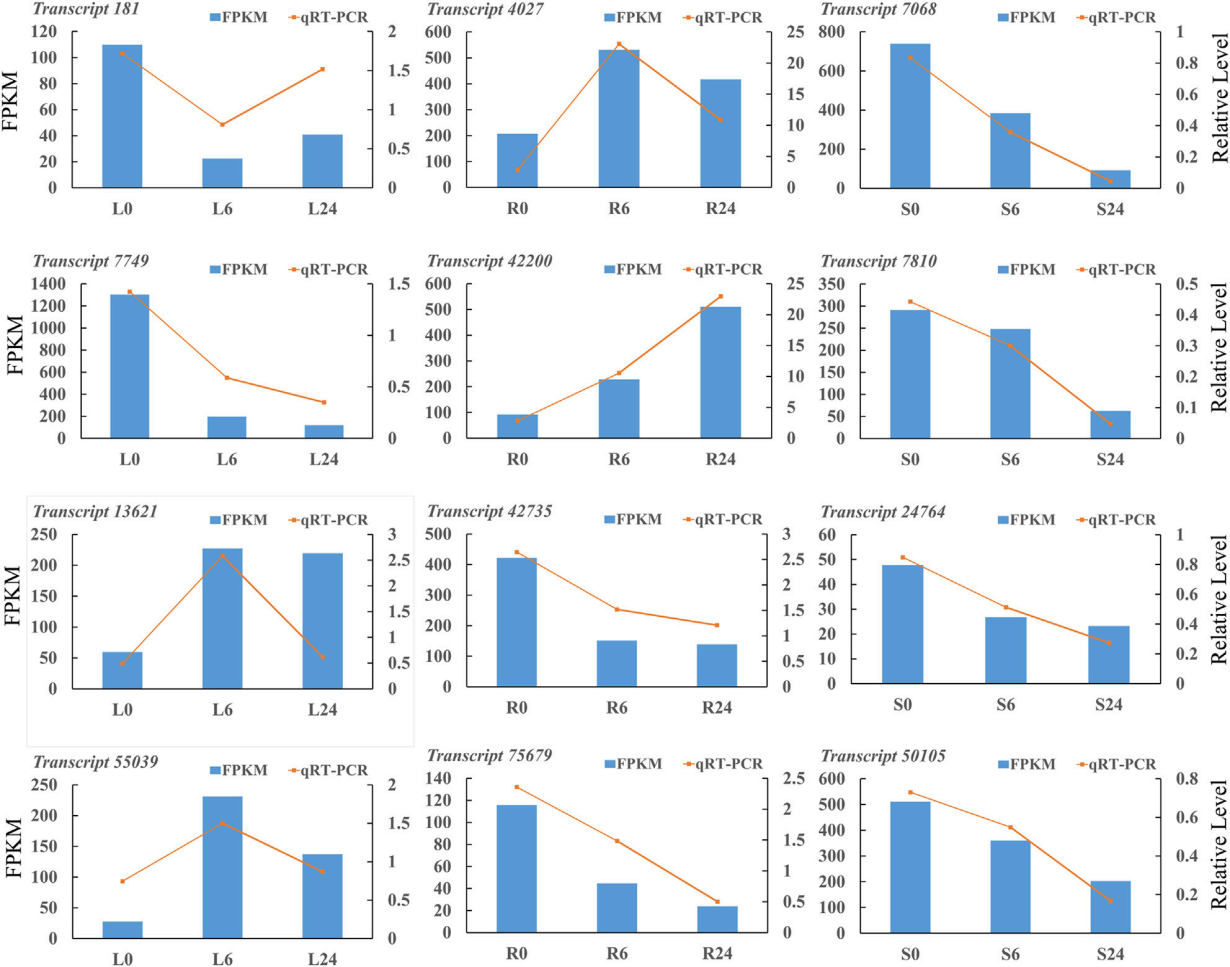
The qRT-PCR analyses of DEGs in *N. tangutorum*. The left vertical axis resents RNA-seq FPKM, the right vertical axis represents qRT-PCR relative expression, and the horizontal axis represents different samples (*transcript 181*: hypothetical protein CISIN_1g000438mg; *transcript 7,749*: hypothetical protein G4B88_011110; *transcript 13,621*: HIPP 24 heavy metal-associated isoprenylated plant protein 24; *transcript 55,039*: auxin-induced protein 22D-like; *transcript 4,027*: berberine bridge enzyme-like 13; *transcript 42,200*: glutathione S-transferase L3-like isoform X1; *transcript 42,735*: xyloglucan endotransglycosylase 3; *transcript 75,679*: nucleotide pyrophosphatase; *transcript 7,068*: protein TIFY 9 isoform X2; *transcript 7,810*: tify domain-containing protein; *transcript 24,764*: Auxin responsive SAUR protein; *transcript 50,105*: transcription factor MYC2-like).

The RNA-seq results were confirmed by performing qRT-PCR experiments. A total of twelve DEGs were selected randomly in three tissues for the validation. The results showed that the relative expression results obtained through qRT-PCR were consistent with the FPKM values provided by Illumina sequencing (Figure 11). The primer sequences of the twelve DEGs are listed in Supplementary Table S13.

## 4 Discussion

*N. tangutorum* is a plant with excellent survival ability in harsh desert environment and has a significant impact on maintaining ecological balance, as its dynamic gene expression regulation network and response mechanism in whole plant level under drought stress remain unexplored, unraveling the drought resistance mechanism of *N. tangutorum* is significant for resistance breeding. The interplay between tissue-common and tissue-specific pathways and genes is crucial for combating drought stress in *N. tangutorum*. We combined second-generation sequencing and third-generation sequencing technologies to comprehensively profile global gene expression across different tissues and time periods of *N. tangutorum* subjected to drought stress, aiming to uncover the genetic background underlying its drought resistance mechanism.

### 4.1 Signaling transduction under drought stress in *N. tangutorum*

Plant growth and development heavily rely on the presence of plant hormones, which also play a crucial role in improving the ability of plants to withstand drought conditions, especially for ABA, ET, BR, and JA (Verma et al., 2016). These hormones play the role of chemical messengers that facilitate the signal transduction process during periods of water scarcity (Ma et al., 2024; Ullah et al., 2018).

ABA is widely recognized as the primary stress hormone crucial for coping with various stress (Ullah et al., 2018). In this study, the core genes, such as *PYL*, *PP2C,* and *ABF* genes were observed in the modulation of ABA signaling. There are more ABA-related DEGs in the roots than in the leaves, and none were found in the stems. This may be due to the fact that ABA is mainly produced in the roots and subsequently transported to the leaves via stems to facilitate physiological regulation like closure stomata (Ullah et al., 2018). Additionally, the expression of *PP2C* genes increased during the drought period except one in leaves, similar results were found in Kentucky Bluegrass and *Phormium tenax* (Bai et al., 2017; Chen et al., 2019). Then *ABF* acted as an important transcription factor downstream of *SnRK2* (sucrose non-fermenting 1-related protein kinase 2) triggering its participation in stomatal regulation (Purayil et al., 2020). By overexpression of *PYL4* in wheat, the possibility of improving drought tolerance lies in reducing transpiration rates while simultaneously increasing photosynthetic activity (Mega et al., 2019). ET in plants exhibits a positive response to abiotic stress. In the current study, The *ETR*, *EBF1/2*, and *EIN2* genes exhibited contrasting trends between leaf and root tissues. This indicates that the ET signal transduction mediated by drought stress show significant disparities in leaves and roots. Previous studies have revealed that the ET production is disturbed in leaves and flower buds, but has almost no effect on root under drought stress in coffee plants (Lima et al., 2021). The findings suggest that the production and signaling of ET in response to drought exhibit tissue-specific patterns. BRs play a crucial role in regulating plant development and their ability to cope with environmental stress. Previous research has indicated a negative effect of *BRI1* on drought tolerance in *Brachypodium distachyon* and tomato (Feng et al., 2015; Nie et al., 2019), which was consistent in our result that all expression changes of *BRI1* were downregulation in all tissues under drought stress. *BKI1* acts as a suppressor, impeding the activation of *BRI1*, which in line with our observation of an upregulation in *BKI1* gene expression and a downregulation in *BRI1* in roots. The pivotal regulators in the JA signaling pathway are exemplified by the protein JAZ and TF *MYC2*, which occupy a central position. In our study, the expressions of both *JAZ* and *MYC2* members were found to be downregulated. Notably, unlike other hormones, alterations in their expression were also observed in stem tissue. Previous studies have indicated that JAZ proteins function not only acts as repressor protein in JA signaling, but also serve as signaling hubs in hormone crosstalk (Wager and Browse, 2012).

The MAPK cascade is a crucial pathway for transmitting signals in plants, which enables the conversion of external stimuli like drought, cold and heat into internal responses. It consists of three crucial kinases: MAPKs (MPKs), MAPK kinases (MAPKKs, MAP2Ks or MEKs) and MAPK kinase kinases (MAPKKKs, MAP3Ks or MEKKs) (Zhang et al., 2021). In our study, the involvement of *MPK3/6*, *MEKK1*, *MAP3K17/18,* and *ANP1* was observed in the transmission of signal under drought-induce conditions. The stress signaling hubs in other plants are primarily represented by the *Arabidopsis* MAP Kinases *MPK6* and *MPK3,* which play crucial role in stomatal development (Hann et al., 2024; Pitzschke et al., 2009). Additionally, the abiotic stress-induced production of ROS leads to the activation of *MPK3/6* (Jalmi and Sinha, 2015). The overexpression of *TaMPK3* resulted in decreased drought tolerance (Liu Y. et al., 2022). On the contrary, the transformation of the *MPK3* gene from cotton into *Arabidopsis* resulted in an improvement in abiotic stress resistance in transgenic plants (Sadau et al., 2021). The *MPK3/6* showed upregulation in leaves after 6 h drought stress, while exhibited downregulation following 24 h drought stress in leaves and roots. In addition, the expression trend of *MAP3K17/18* showed opposite patterns among different genotypes in broomcorn millet (Yuan et al., 2022). These results indicate that the components of MAPK cascades in different species have varying roles under drought stress, thus further research is needed to investigate the role of MAPK members in *N. tangutorum*.

### 4.2 Enzymes and DEGs related to ROS scavenging in *N. tangutorum*

Drought could cause higher production of ROS, resulting in the destruction of essential macromolecules, disturbance of the cellular redox equilibrium, the breakdown of chlorophyll and oxidative damage to membranes (Kumar et al., 2021). The MDA content increased 24 h after the induction of drought stress by PEG (Figure 9B), indicating that drought stress caused peroxidation of membrane lipid. Plants have evolved a protective mechanism that involves the enzymes SOD, POD, glutathione S-transferase (GST) and CAT to eliminate excess ROS (He et al., 2017). SOD is believed to catalyze the O_2_^−^ into H_2_O_2_, while CAT and POD decompose H_2_O_2_ (Tian et al., 2012). In addition, GST is also a crucial enzymes member in ROS defense system (Yan et al., 2008). These antioxidant enzymes work synergistically to detoxify ROS (Sarker and Oba, 2018). In this study, the H_2_O_2_ content change varied in different tissues with prolonged drought stress, while the pattern of antioxidative enzymes showed inconsistency among the three tissues under different drought treatments time points. Correspondingly, the regulation of *SOD*, *CAT*, *GST,* and *POD* genes in *N. tangutorum* showed variations in expression levels when subjected to drought stress. The collaboration between antioxidant enzymes and associated DEGs ensures the equilibrium between ROS generation and detoxification, thereby safeguarding plants against oxidative stress caused by ROS.

### 4.3 The roles of drought-responsive pathways and metabolites in *N. tangutorum*

Drought stress not only induces the build-up of sugars and adjusts energy demands in plants but also facilitates the conversion of storage sugars (such as starch) into soluble forms to decrease cell water potential (Kaur et al., 2021). It has been established that the regulation of plant carbon metabolism is associated with the activities of sucrose synthase and alpha-amylase under drought stress (Min et al., 2020). Sucrose synthase (SuS) metabolizes sucrose into hexose phosphates, which are precursor for starch biosynthesis. Alpha-amylase (AMY) is a key enzyme related to the decomposition of starch and belong to the family 13 of glycosyl hydrolase (Dong and Beckles, 2019; Janecek, 1997). It was noticed that the expression of *SuS* and *AMY* genes exhibited differential regulation in leaves under drought stress. Additionally, the soluble sugar content increased at 6 h and decreased at 24 h in whole plant, suggesting that the starch and sugar conversion was dynamic and complex in *N. tangutorum* due to its dual role as an energy source and osmotic regulator for drought resistance, the ratio of cellular sugars and starch can be changed by stress-induced regulation of starch metabolism (Dong and Beckles, 2019). These finding implies that the drought resistance in *N. tangutorum* is linked with carbon metabolism.

The lignin pathways represent a prominent branch of the phenylpropanoid metabolism, and lignin play a role as mechanical support to cell wall and contributes to plant responses to drought stress by facilitating cell wall thickening, which reduces water permeability and maintains the plant cell turgor. The *CAD* and *4CL* genes are all key enzyme genes encoding lignin synthesis (Dong and Lin, 2021). Upregulation of *CAD* gene in oriental melon can induce the accumulation of lignin and respond actively to drought stress (Liu et al., 2020; Silva et al., 2018). The *Gh4CL7* gene of cotton plays a crucial role in the biosynthesis of lignin and confers enhanced drought resistance (Sun et al., 2020). During the process of lignin formation, peroxidases function as catalysts that polymerize monolignols into lignin (Dong and Lin, 2021). In current study, the upregulation of all 4*CL* and *CAD* genes and significantly higher activity of POD in roots indicate that the crucial role of lignin in drought resistance in *N. tangutorum*.

Amino acids serve as osmoprotectants in reaction to abiotic environmental factors (Batista-Silva, et al., 2019). In our study, the expression of 7 *P5CS* genes was upregulated in root tissues after 24 h of drought, while the expression of 2 genes encoding proline dehydrogenase (*PRODH*), a pivotal enzyme involved in proline degradation (Kamanga et al., 2018), were significantly downregulated in all three tissues after 24 h drought stress. These results contribute to an increase in proline content, which in consistent with the physiological tests showing increased proline content in leaves, stems and roots of *N. tangutorum* following 24 h of drought treatment. Furthermore, these findings highlighted the active response of *PRODH* to drought stress after 24 h. In addition to *P5CS*, the upregulation of a majority of genes in “biosynthesis of amino acids” pathway implies that amino acids may play additional roles in enhancing drought resistance of *N. tangutorum*. During the process of resisting drought in sugarcane, amino acid metabolism fulfilled dual function by signaling and providing carbon skeletons for energetic maintenance (Contiliani et al., 2022; Diniz et al., 2020).

### 4.4 Transcription factors play a vital role under drought stress in *N. tangutorum*

TFs act as the activation switch for cascades of signal transduction by recognizing the particular cis-elements located in promoter region of their target genes (Franco-Zorrilla et al., 2014). As regulatory genes, TFs have a significant impact on the regulation of numerous genes associated with the response to stress (Joshi et al., 2016; Singh and Laxmi, 2015). In our study, we observed significant alterations in the expression of *AP2/ERF*, *WRKY*, *NAC*, *bHLH*, and *MYB* family members following exposure to drought stress. The family of *AP2/ERF* is exclusive to plants that covers a large number of members in common plant species (e.g., 145 in *Arabidopsis*, 170 in rice, 200 in poplar and 291 in Chinese cabbage), playing a part in the ethylene-responsive transcription (Joshi et al., 2016). *AP2/ERF-ERF* as a subfamily belonging to the *AP2/ERF* superfamily, and the TFs from the *ERF* subfamily are considered tightly associated with responding to abiotic stresses and involved in hormone signaling pathway (Li et al., 2017; Mizoi et al., 2012). This finding aligns with our research results, indicating that the “plant hormone signal transduction” pathway exhibits a remarkably high degree of activity and there are numerous *AP2/ERF-ERF* TFs, which indicated that *AP2/ERF-ERF* subfamily significantly contributes to enhancing the ability of *N. tangutorum* to resist drought. The *WRKY* TFs take an active part as prominent TFs, similar to *AP2/ERF-ERF*, the two TFs constitute a quarter of the different expressed TFs, highlighting their important role as viable options for improving resistance to drought in *N. tangutorum*. *WRKY* transcription factors exhibited tissue-specific expression patterns. In leaf tissues, 4 out of 5 *WRKY*s showed downregulation followed by upregulation after 6 h and 24 h of drought stress, while in root tissues, differential regulation occurred for 6 out of 7 *WRKYs* after 6 h and downregulation was observed after 24 h. These results may suggest that *WRKY* members were involved in various biological processes in different tissues to respond to drought in *N. tangutorum*. *MYB* TFs contribute to the improvement of plant resilience against drought by controlling the activity of genes associated with metabolite production and stomatal response (Wang X. et al., 2021). And *MYB* TFs were implicated in the drought response of *N. tangutorum* across three distinct tissues.

It has been shown that the collaboration among TFs in drought response is crucial for the regulation of downstream gene expression (Singh and Laxmi, 2015). In the current study, more than 200 TFs altered their expression under drought stress. These master regulatory genes, TF families, hold great potential for mitigating drought stress, and overexpression these TFs through genetic engineering approaches can be employed to develop the stress-tolerant crops (Joshi et al., 2016; Shao et al., 2015). Additionally, *N. tangutorum* may serve as a valuable gene bank for breeding purposes.

### 4.5 Analysis of hub genes in response to drought in *N. tangutorum*

WGCNA analysis is a powerful tool for identifying hub genes by exploring the correlation between the gene module with similar expression patterns and physiological indexes (Zhu et al., 2024). In our study, the cyan module revealed *PP2C*, *ACO*, *GST*, and *GAPDH* genes as well as *AP2/ERF*, *WRKY*, *MYB* TFs to be identified as hub genes. *PP2Cs* plays a negative regulatory role in ABA signaling pathway (Mega et al., 2019), while *ACO* encodes the key enzyme in ET biosynthesis (Tang and Woodson, 1996), both were involved in the hormone regulation. The identification of two *GST* genes as hub genes further emphasizes the pivotal role in ROS scavenging for the drought resistance of *N. tangutorum*. *GAPDH* acts as an enzyme that facilitates the oxidation of triose phosphate in the process of glycolysis (Hildebrandt et al., 2015). It has been found that *GAPDH* also serves as a hub gene in enhancing drought tolerance by participating in photosynthetic adaptation of rice (Chintakovid et al., 2017). Despite being commonly used as a reference gene in transcriptomic and proteomic analyses, the upregulated expression of *GAPDH* genes during drought stress in *N. tangutorum* suggests the potential active response to drought stress through glycolysis process. The *AP2/ERF*, *WRKY,* and *MYB* TFs are closely associated with the regulation of downstream drought-responsive genes. The WGCNA results indicate that ABA and ET regulation, ROS scavenging, glycolysis are fundamental strategies employed by *N. tangutorum* to cope with drought.

### 4.6 Schematic model of the response to drought stress in *N. tangutorum*

**FIGURE 12:**
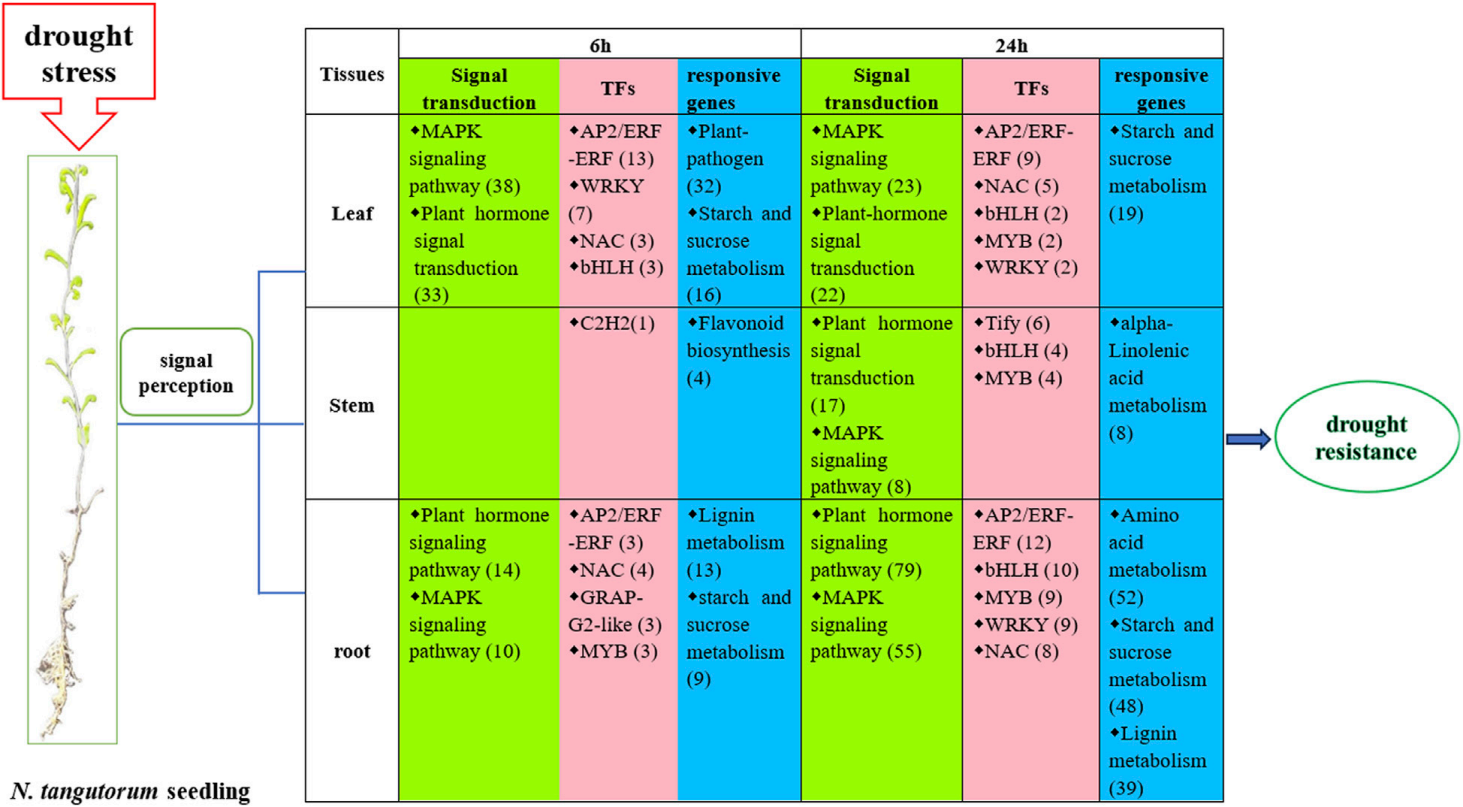
A schematic model of the transcriptional regulatory process in response to 6 h and 24 h drought stress in *N. tangutorum* at the whole-plant level (The numbers in bracket mean the number of related DEGs).

We proposed a hypothetical model of drought responses in *N. tangutorum* based on the previous pathway and DEGs analysis (Figure 12). When plant suffer from drought stress, plant cell membranes are equipped with the ability to sense stress signals (Mahmood et al., 2020). In our study, we found that the top two ways of drought signaling in roots and leaves were based on hormones and MAPK components after 6 h and 24 h of drought stress. The same response was observed in stems after 24 h of drought stress. The DEGs related to hormone signaling involved eight types of plant hormones, IAA, GA, CTK, ABA, ET, BR, SA, and JA in leaves and roots at both investigated time point, which indicates that hormones are crucial in enabling *N. tangutorum* to endure stress caused by drought. Entire cascades of stress signal were transmitted in the nucleus, and TFs were activated to regulate a cluster of target genes. The *AP2/ERF-ERF, WRKY, NAC, bHLH, MYB* TFs are the main force in stress management in roots and leaves, while *Tify* TF play a principal role in stems after 24 h drought stress. These TFs interact with promoter regions of genes that exhibit sensitivity to stress, which involved in minimization of oxidative damage, metabolism adjustment (sugar, lignin and amino acid) and plant-pathogen interaction, upregulation or downregulation of these genes enhanced combinatorial tolerance against drought stress. In the process, leaves and roots share more common signal transduction pathway and TF, while stems have respective pathway with fewer DEGs, which may due to stem take an active part in integrating message from the roots and aerial part of *N. tangutorum* for its vascular system (Takahashi et al., 2020). In general, different tissues of *N. tangutorum* respond synergistically to drought with tissue-specific function, contributing to harmonization of plant tissues and overall stress tolerance on whole-plant level.

## 5 Conclusion

In this research, we investigated the response how *N. tangutorum* reacts to drought by conducting bioinformatics analysis and measuring physiological indexes. We examined three parts of the whole plant (leaves, roots and stems) and integrated Illumina RNA-seq and PacBio SMRT-seq to obtain comprehensive transcripts of *N. tangutorum*. The analysis of key pathways and DEGs revealed a synergistic modulation in response to the adverse effects of drought stress, *N. tangutorum* relied on hormones and the MAPK cascade for transmitting and responding to stress signals at the whole-plant level. Both leaf tissue and root tissue enhanced drought tolerance through carbon metabolism, while root tissue also employed lignin synthesis and amino acid metabolism, enabling *N. tangutorum* to effectively cope with unfavorable conditions. Furthermore, TFs analysis showed that *AP2/ERF-ERF*, *WRKY*, *bHLH*, *NAC*, *MYB* actively participated in responding to the stress induced by drought. The physiological indexes demonstrated the drought-induced physiological response of *N. tangutorum*. The WGCNA analysis revealed a candidate hub gene network related to drought stress. The results provide a basis for further exploration into the regulatory mechanisms involved in *N. tangutorum*’s adaptation to drought stress.

## Data Availability

The datasets presented in this study can be found in online repositories. The names of the repository/repositories and accession number(s) can be found below: https://www.ncbi.nlm.nih.gov/sra/PRJNA1094917.
