# Supplementary material for: Integrated PacBio SMRT and Illumina sequencing uncovers transcriptional and physiological responses to drought stress in whole-plant Nitraria tangutorum
Source: Front Genet. 2024 Oct 1;15:1474259. doi: 10.3389/fgene.2024.1474259 (PMC11473341; doi:10.3389/fgene.2024.1474259)
Supplement: Supplementary file 2 [file DataSheet1.docx]

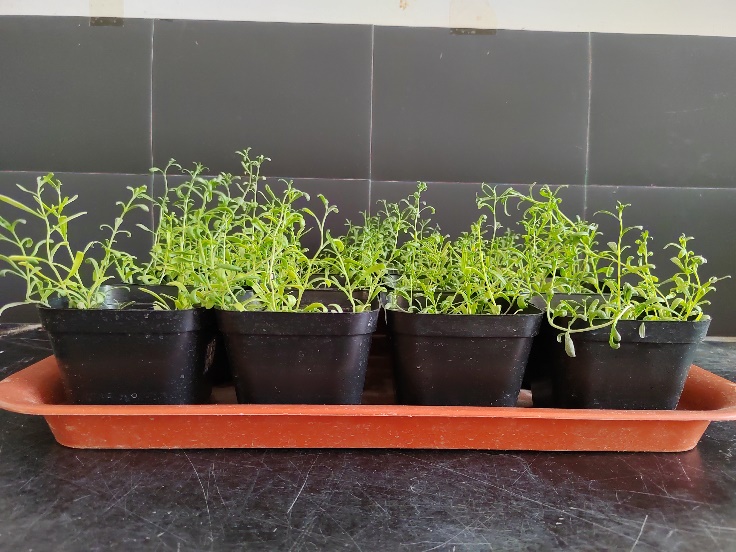

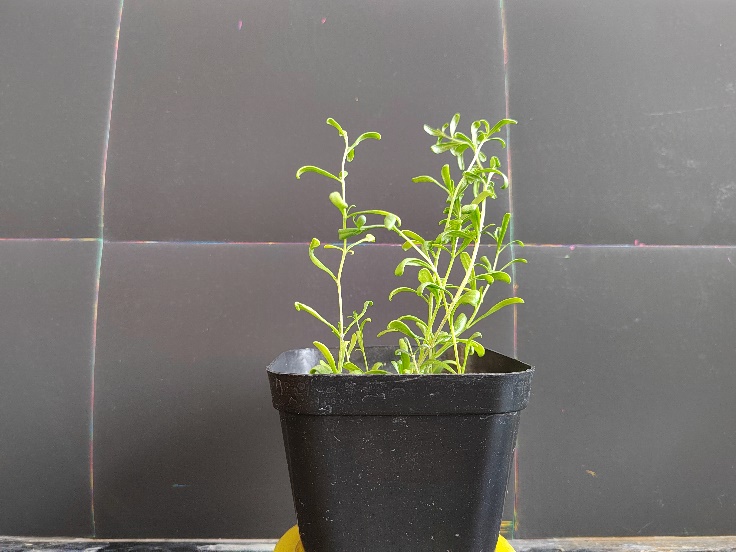


**
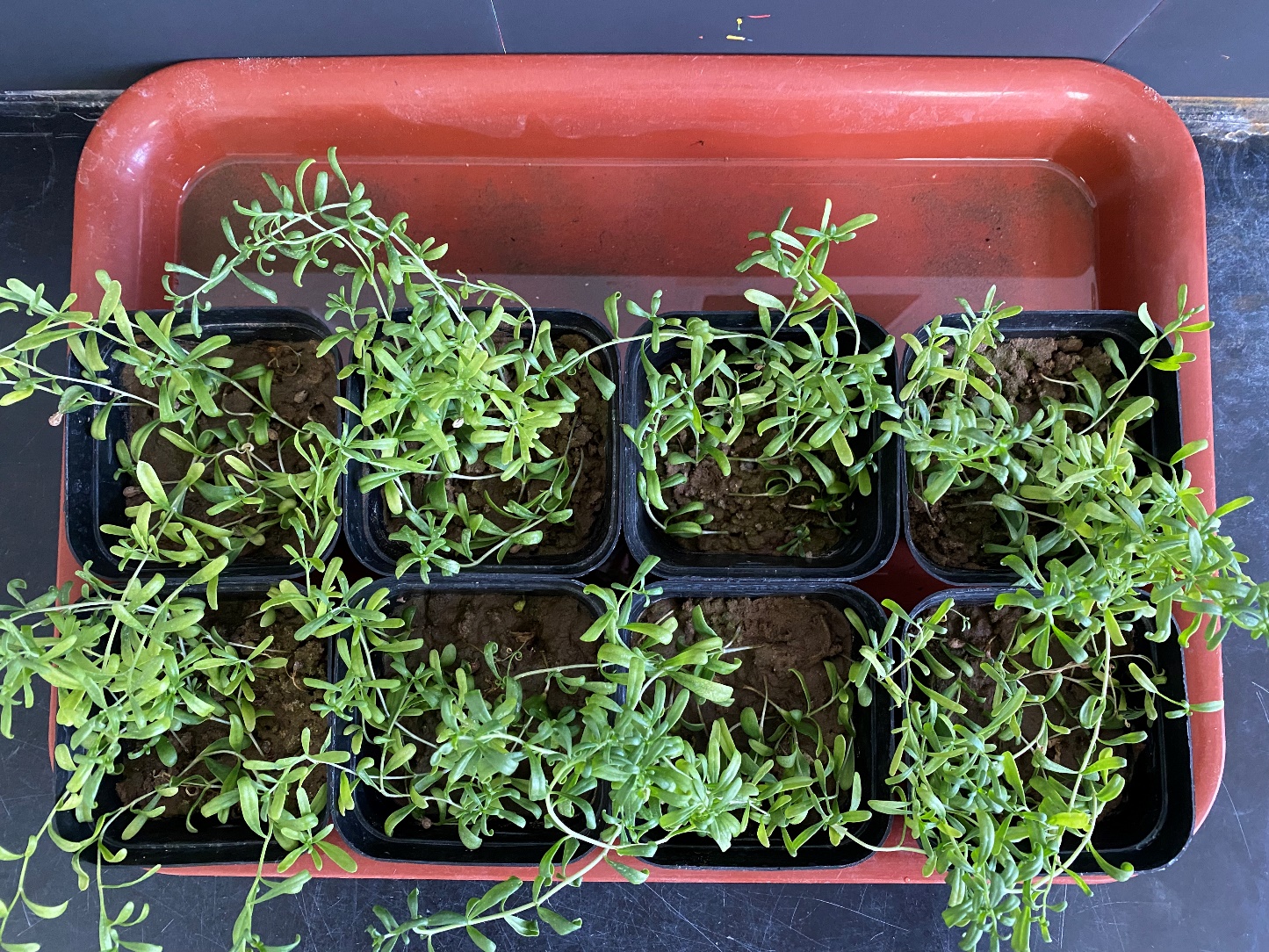
Supplementary Figure S1**. The *N. tangutorum* seedlings before PEG treatment (0h).

**Supplementary Figure S2**. The *N. tangutorum* seedlings were subjected to a 6h treatment with PEG.


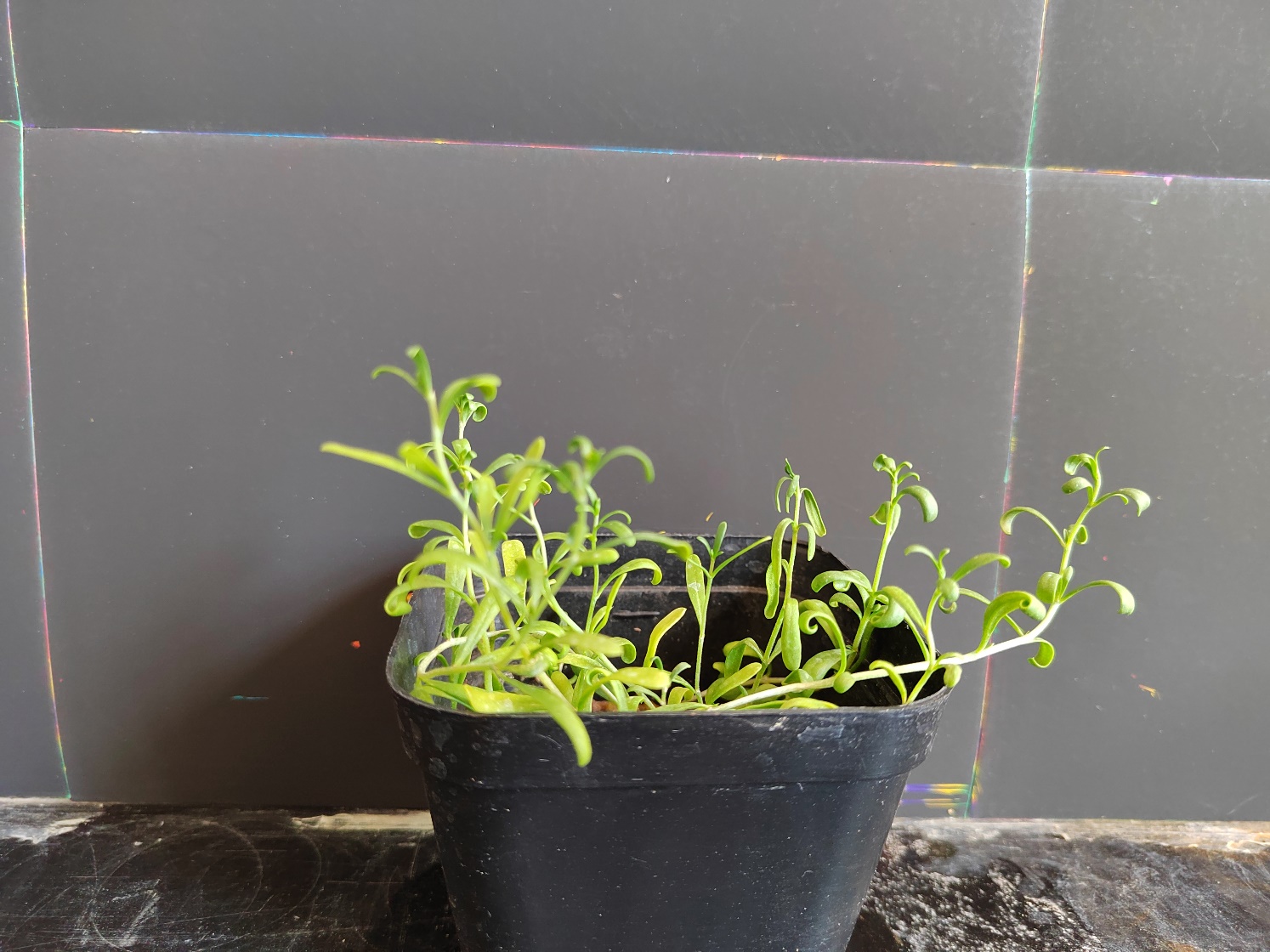


**Supplementary Figure S3**. The *N. tangutorum* seedlings were subjected to a 24 treatment with PEG.


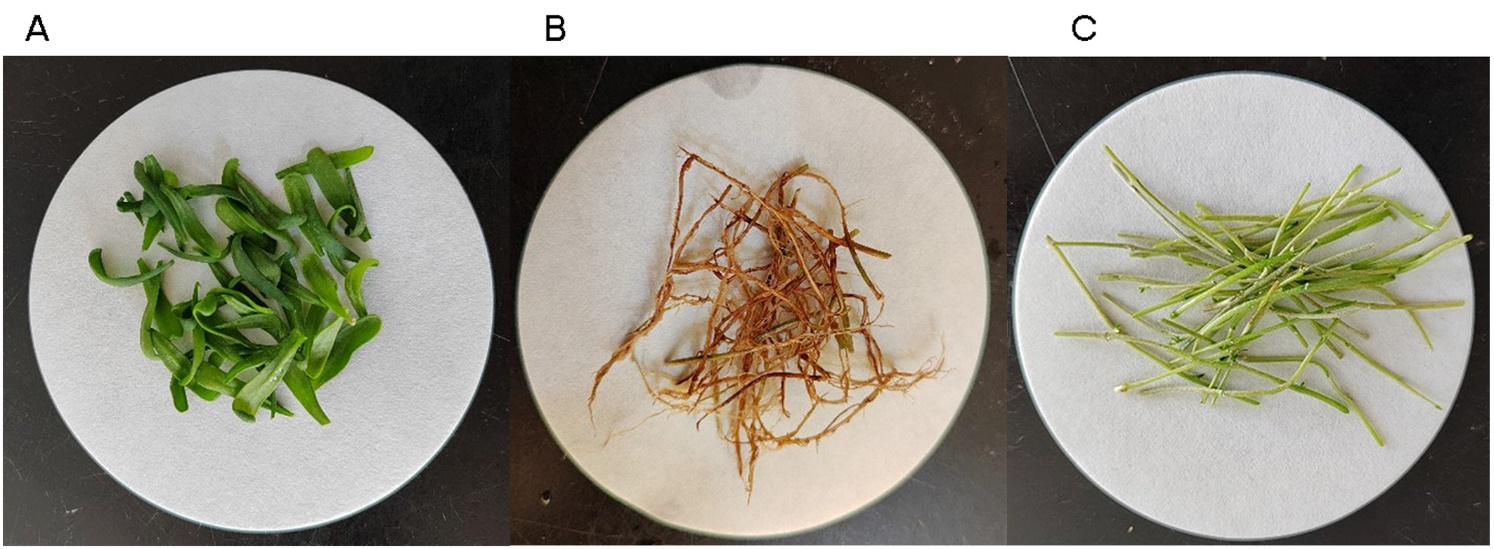


**Supplementary Figure S4**. The sample of leaves, roots and stems of *N. tangutorum.* (**A**) Leaf samples. (**B**) Root samples. (**C**) Stem samples.


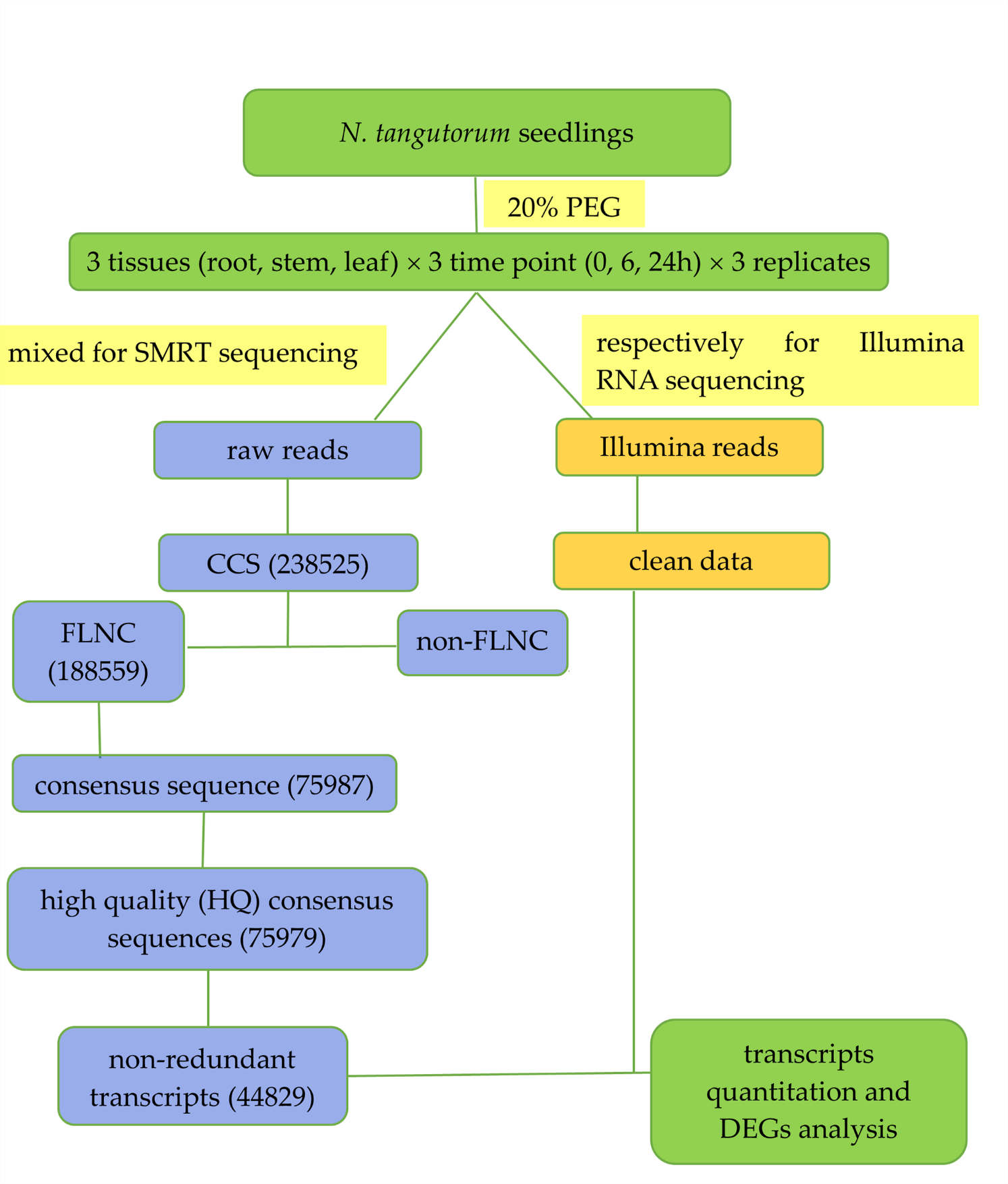


**Supplementary Figure S5**. Overview of the sequence data process.


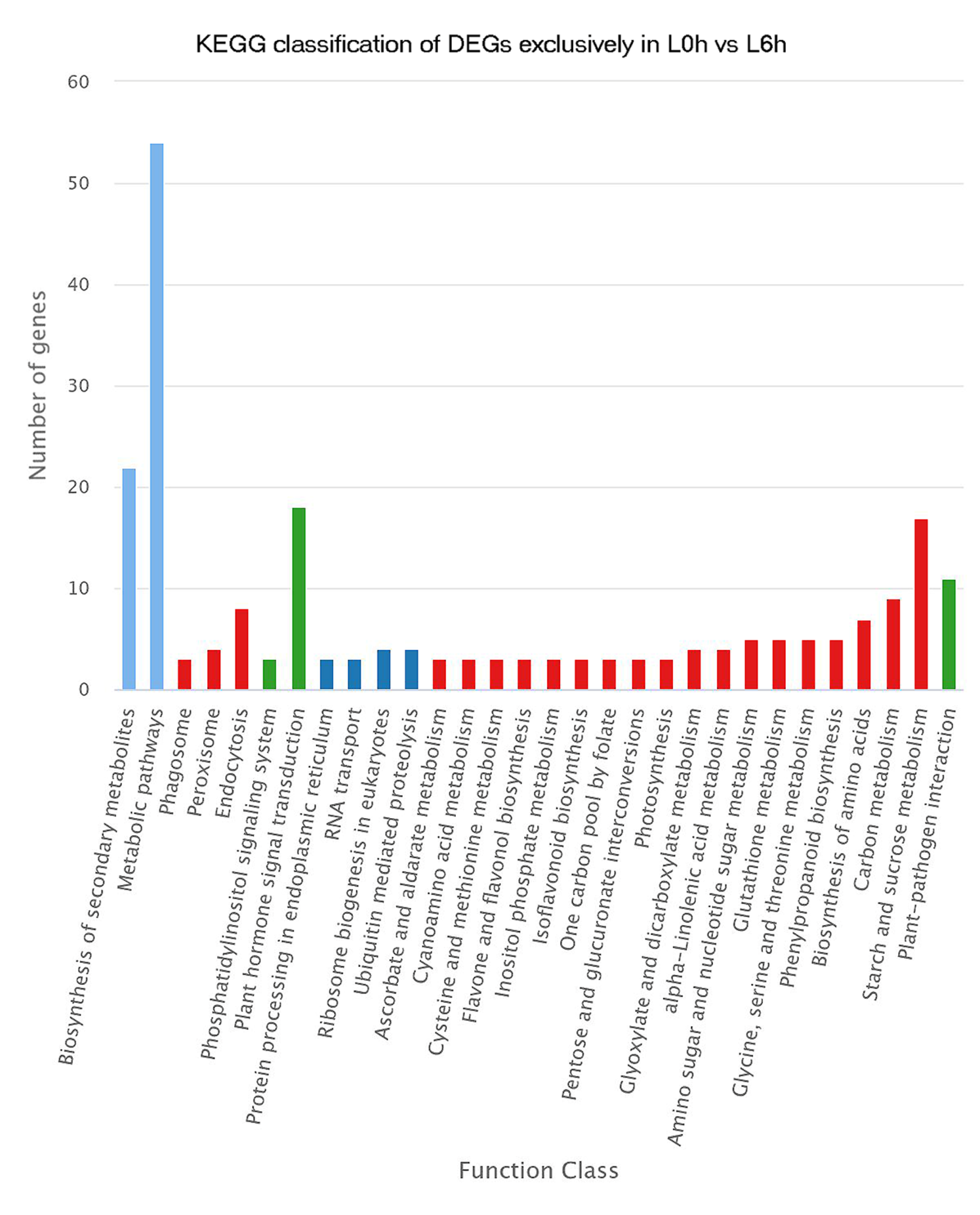
**Supplementary Figure S6. KEGG classification of DEGs exclusively in L0h vs L6h.**


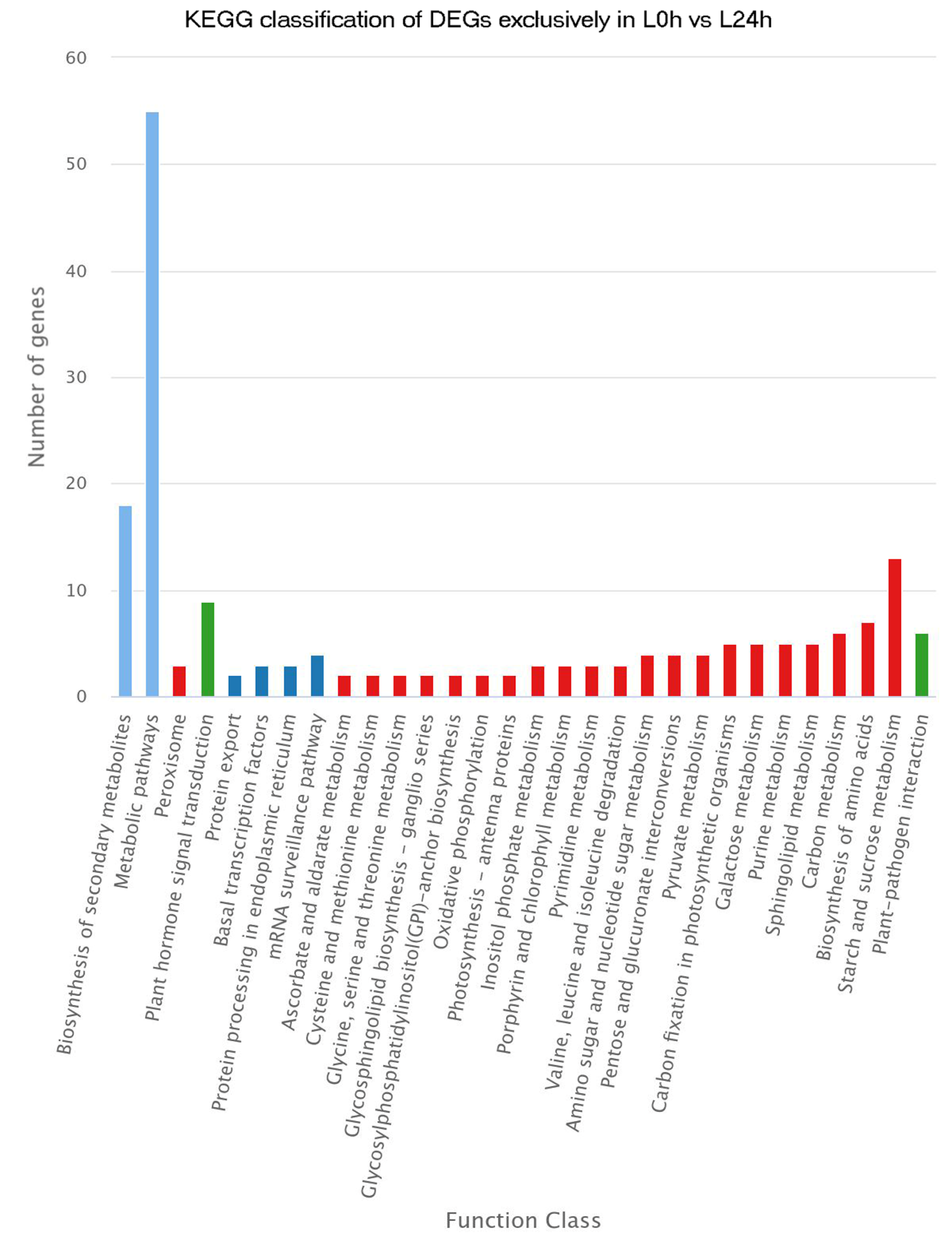
**Supplementary Figure S7. KEGG classification of DEGs exclusively in L0h vs L24h.**


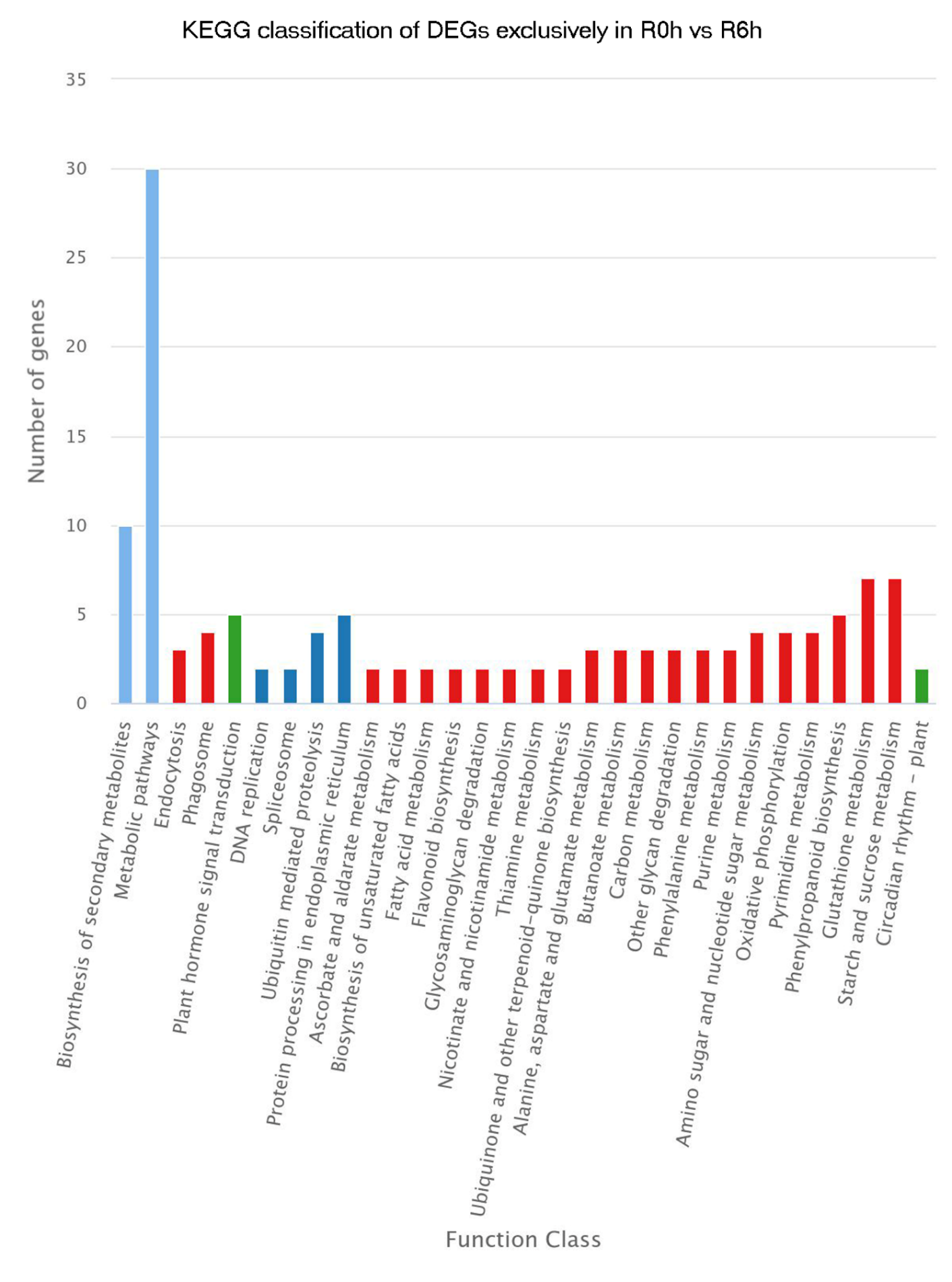
 **Supplementary Figure S8. KEGG classification of DEGs exclusively in R0h vs R6h.**


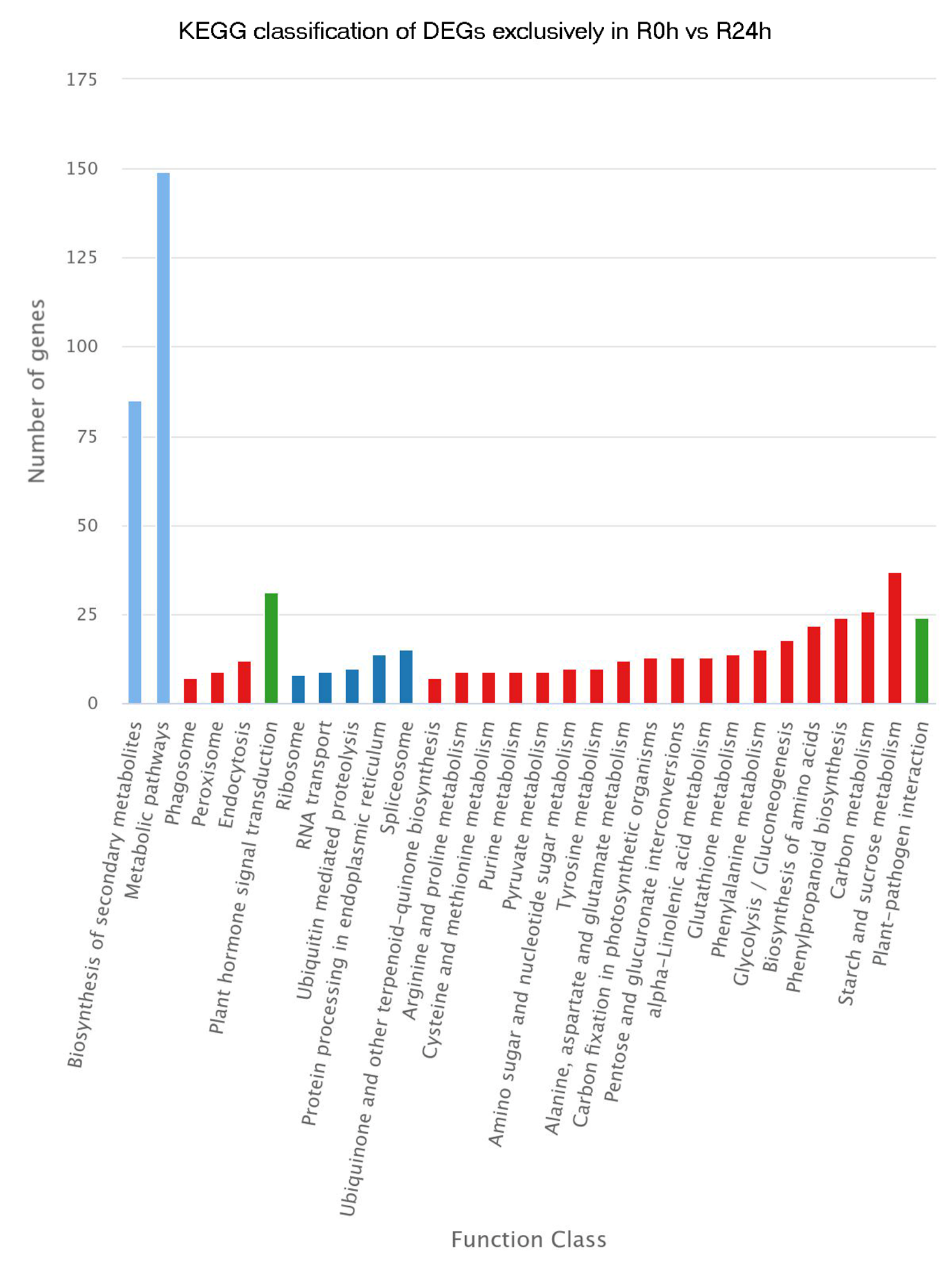
 **Supplementary Figure S9. KEGG classification of DEGs exclusively in R0h vs R24h.**


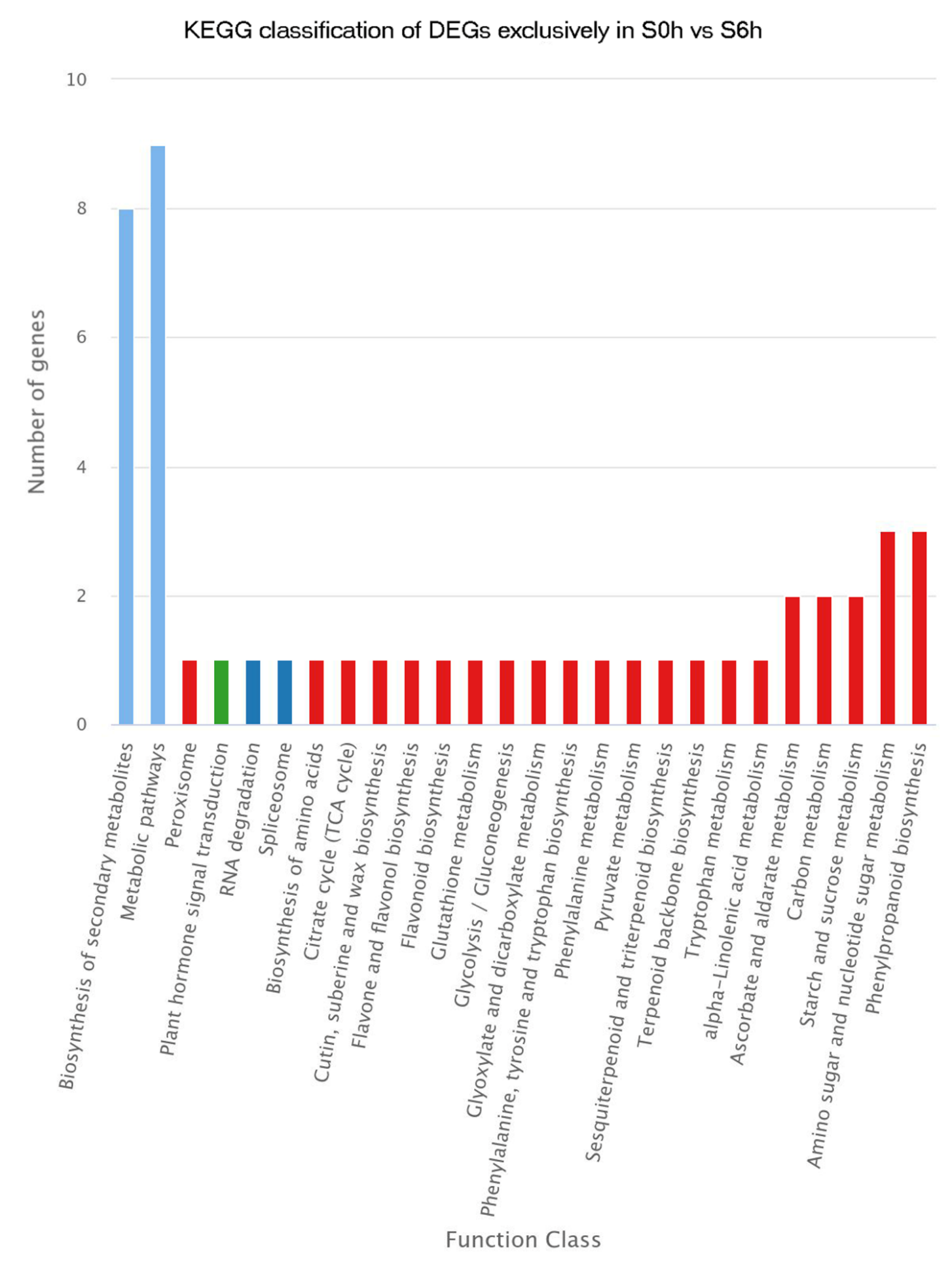
 **Supplementary Figure S10. KEGG classification of DEGs exclusively in S0h vs S6h.**


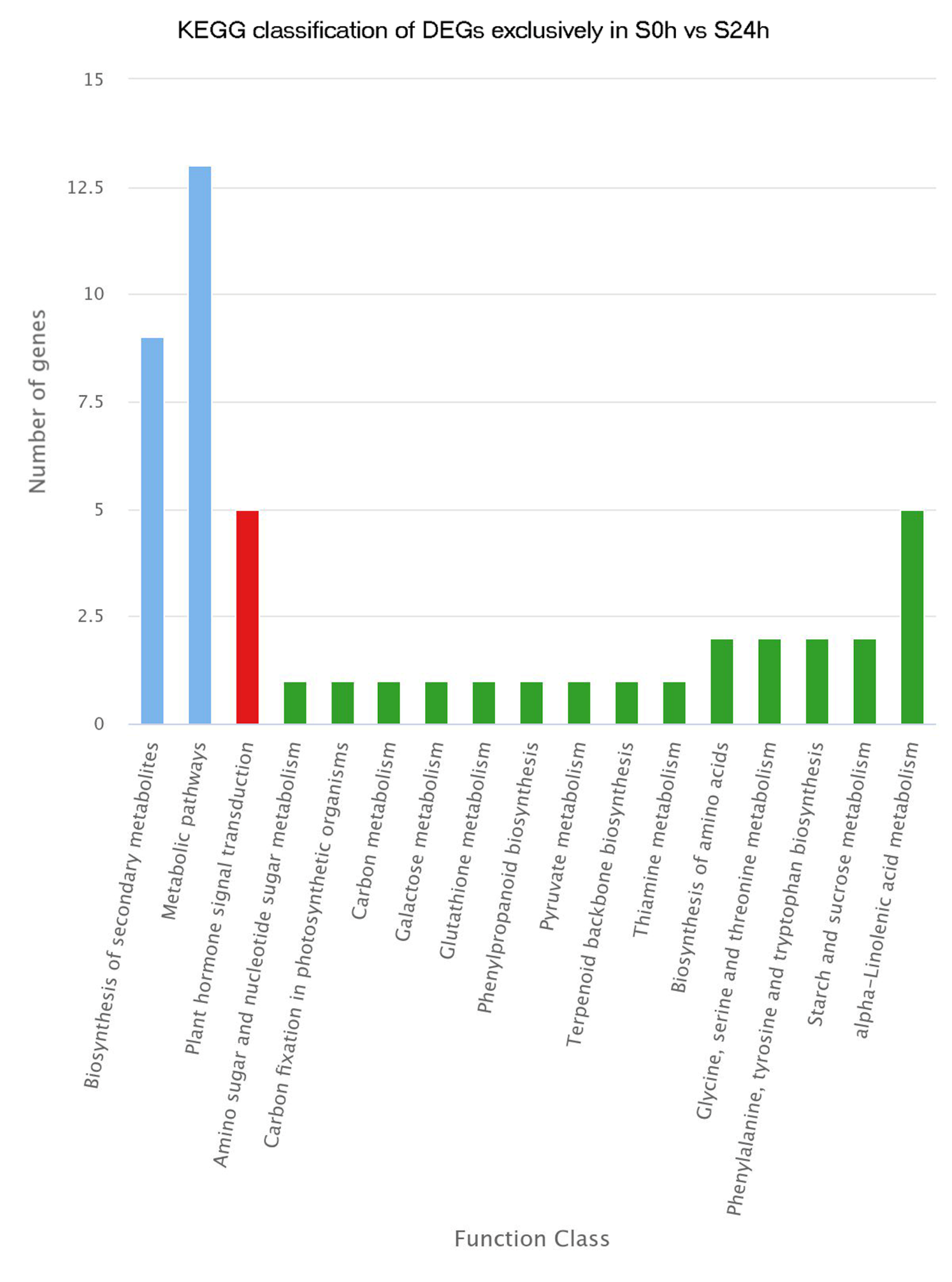
 **Supplementary Figure S11. KEGG classification of DEGs exclusively in S0h vs S24h.**
